# Approaching the Spin-Statistical Limit in Visible-to-Ultraviolet Photon Upconversion

**DOI:** 10.1021/jacs.1c13222

**Published:** 2022-02-17

**Authors:** Axel Olesund, Jessica Johnsson, Fredrik Edhborg, Shima Ghasemi, Kasper Moth-Poulsen, Bo Albinsson

**Affiliations:** † Department of Chemistry and Chemical Engineering, Chalmers University of Technology, 41296 Gothenburg, Sweden; ‡ Institute of Materials Science of Barcelona, ICMAB-CSIC, Bellaterra, 08193 Barcelona, Spain; § Catalan Institution for Research and Advanced Studies ICREA, Pg. Lluís Companys 23, 08010 Barcelona, Spain

## Abstract

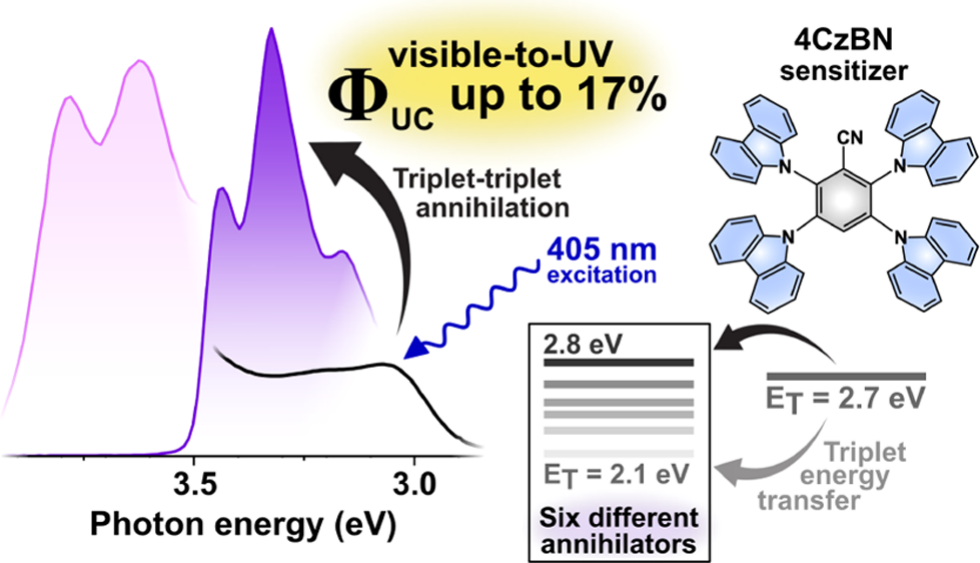

Triplet–triplet annihilation photon upconversion (TTA-UC) is a process in which triplet excitons combine to form emissive singlets and holds great promise in biological applications and for improving the spectral match in solar energy conversion. While high TTA-UC quantum yields have been reported for, for example, red-to-green TTA-UC systems, there are only a few examples of visible-to-ultraviolet (UV) transformations in which the quantum yield reaches 10%. In this study, we investigate the performance of six annihilators when paired with the sensitizer 2,3,5,6-tetra(9*H*-carbazol-9-yl)benzonitrile (4CzBN), a purely organic compound that exhibits thermally activated delayed fluorescence. We report a record-setting internal TTA-UC quantum yield (Φ_UC,g_) of 16.8% (out of a 50% maximum) for 1,4-bis((triisopropylsilyl)ethynyl)naphthalene, demonstrating the first example of a visible-to-UV TTA-UC system approaching the classical spin-statistical limit of 20%. Three other annihilators, of which 2,5-diphenylfuran has never been used for TTA-UC previously, also showed impressive performances with Φ_UC,g_ above 12%. In addition, a new method to determine the rate constant of TTA is proposed, in which only time-resolved emission measurements are needed, circumventing the need for more challenging transient absorption measurements. The results reported herein represent an important step toward highly efficient visible-to-UV TTA-UC systems that hold great potential for driving high-energy photochemical reactions.

## Introduction

Unconventional strategies for expanding the use of solar energy have attracted significant attention in recent years.^1,2^ Using photon upconversion (UC), in which low-energy photons are combined to form high-energy light, it is expected that the conventional limits in photovoltaics can be shifted upward.^3^ This process has also been utilized in contexts of, for example, optogenetics,^4,5^ targeted drug-delivery,^6^ photocatalysis,^7^ and photochemistry.^8,9^

For solar applications, the mechanism called triplet–triplet annihilation photon UC (TTA-UC) is of specific interest as this process functions under low-intensity, noncoherent light.^10,11^ By using a donor, or sensitizer, species in conjunction with a fluorescent annihilator, triplets generated by the sensitizer from incident long-wavelength light may be converted into a highly energetic singlet state within the annihilator species in a spin-allowed TTA process. This scheme has been demonstrated for many different spectral ranges and with a variety of compounds,^12^ spanning purely organic systems,^13,14^ nanocrystals,^15−19^ metallic complexes,^20−24^ and metal–organic frameworks,^25^ to name a few.

The most success in terms of UC efficiencies has been obtained in the visible region. In particular, red-to-blue TTA-UC systems have been reported with UC quantum yields (Φ_UC_) as high as 42% (out of a theoretical maximum of 50% owing to the two-to-one nature of the UC process),^26^ while other spectral regions have proven more challenging.^a^ Upconverting near-infrared or infrared light to the visible region, which is especially important for biological and photovoltaic applications, have seen much lower efficiencies with a Φ_UC_ of 8% at best.^27^ Similarly, the performance of visible-to-ultraviolet (vis-to-UV) TTA-UC systems suffers from limited efficiencies. Significant progress has however been made recently, with reports on a Φ_UC_ of around 10% for three different systems.^28−30^ Still, there is no fundamental reason as to why much higher efficiencies would not be possible.

Only a few UV-emitting species have been employed in TTA-UC to date, with 2,5-diphenyloxazole (PPO) arguably gaining the most attention.^14,30−35^ Pioneering work by the Castellano group dating back to 2009 employed PPO together with biacetyl, albeit with very low efficiencies.^14^ It is only as of 2021 that a system employing PPO surpassed 10% in Φ_UC_, which was achieved by pairing PPO with a cadmium sulfide nanocrystal sensitizer decorated with 3-phenanthrene carboxylic acid.^30^ The 10% limit has also been surpassed by pairing an iridium complex or a ketocoumarin derivative with 1,4-bis((triisopropylsilyl)ethynyl)naphthalene (TIPS-Naph), systems which also demonstrate low threshold excitation intensities (*I*_th_).^28,29^ Other annihilators previously investigated include other naphthalene and oxazole derivatives,^8,30,36^ species from the terphenyl family,^13,37^ and as of recently also a biphenyl derivative with the capability to emit light beyond 4 eV.^38^

The spin-statistical factor, *f*, gives the probability that an excited annihilator triplet state ultimately ends up as a singlet excited state following TTA. In an annihilator species in which the second triplet excited state (T_2_) is energetically accessible during TTA, *f* takes the value of 2/5 for strongly exchange-coupled triplet pairs, which caps the internal Φ_UC_ to 20%.^39^ Annihilators yielding significantly higher Φ_UC_, such as a few based on perylene,^26,40^ have been shown to have *f* ≈ 1 since T_2_ has too high energy to be populated following TTA. This classical way of approaching the spin-statistical factor has recently been questioned, suggesting that a broader range of values could be achieved, which depends on, for example, the nature of the initially formed triplet pair states.^39^

In this study, we aim to shine light on the fundamental aspects currently limiting vis-to-UV TTA-UC. A thorough and systematic investigation of both known, relatively efficient, annihilator species as well as two compounds that have not been used in this context previously has been performed. The six annihilators used here are paired with a high triplet energy thermally activated delayed fluorescence (TADF)-type sensitizer, allowing for efficient population of also highly energetic annihilator triplet states. We show that also vis-to-UV TTA-UC systems may approach the spin-statistical limit of 20%. Specifically, employing TIPS-Naph as the annihilator species yields a record-setting internal Φ_UC_ of 16.8% (out of a 50% maximum), which is a significant improvement on the previously best performing vis-to-UV TTA-UC systems.^28−30^ High internal Φ_UC_ values are also obtained for PPO (14.0%), 2,5-diphenylfuran (PPF, 13.0%), a compound never used for vis-to-UV TTA-UC before, and for *p*-terphenyl (TP, 12.6%), a compound which emits much deeper in the UV region. The performances of the remaining systems are also evaluated, and the intrinsic properties governing the TTA-UC process are obtained and analyzed. Further, we discuss what implications these findings have and what obstacles still need to be overcome in order to improve these systems for future application in photochemical settings.

## Results

### Photophysical Characterization

**Figure 1:**
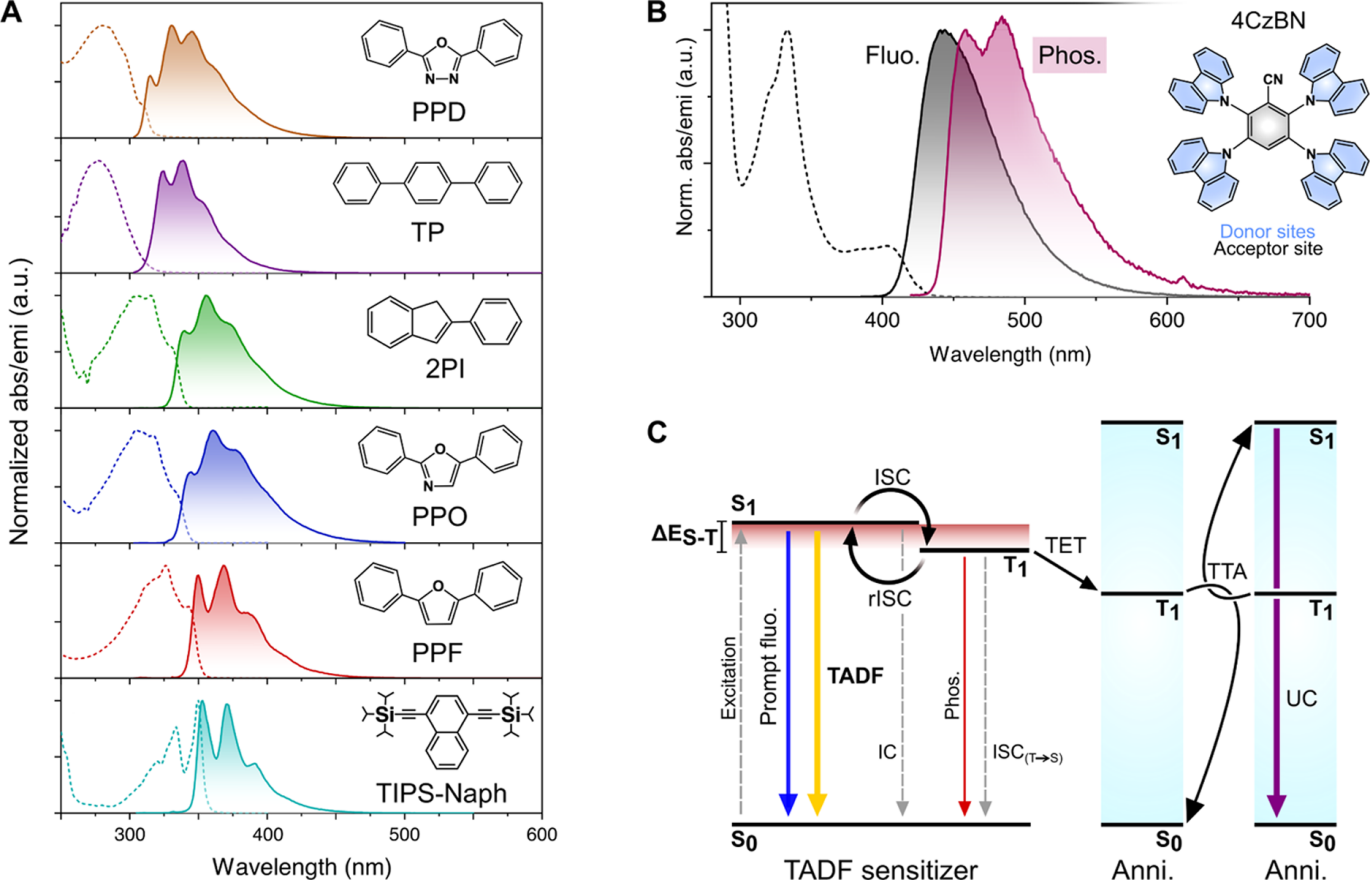
(A) Normalized absorption spectra in THF (dashed) and steady-state fluorescence spectra in toluene (solid) of the investigated annihilators and their respective molecular structure. (B) Normalized absorption (dashed) and steady-state fluorescence (black solid) at 295 K in toluene, normalized phosphorescence (solid purple) at 77 K in 2-methyl-THF (MTHF), and molecular structure of sensitizer 4CzBN. (C) Jablonski diagram depicting the different photophysical processes within a TADF compound alongside the additional steps necessary to afford TTA-UC.

The annihilators under investigation herein are presented in Figure 1A alongside their respective absorption and fluorescence spectra. PPO, TIPS-Naph, TP, and 2,5-diphenyl-1,3,4-oxadiazole (PPD) have all been used for TTA-UC previously, while PPF and 2-phenylindene (2PI), to the best of our knowledge, are demonstrated as annihilators for the first time. These compounds all emit UV light efficiently, albeit with nonunity quantum yields (Table 1), but their respective first singlet and triplet excited state energies are quite different, spanning 3.5–4.0 eV (singlets) and 2.1–2.8 eV (triplets, Table 1). Even though this study is primarily conducted in toluene as the solvent, the absorption spectra in Figure 1A are measured in tetrahydrofuran (THF) since toluene absorption interferes with the spectral shape of annihilator absorption below 290 nm. To make comparison between annihilator species as feasible as possible, we chose to use only one sensitizer. While cadmium sulfide nanocrystals have previously been used to sensitize high triplet energy annihilators such as PPD,^30^ their notoriously complex photophysics,^44^ the need for additional mediating compounds,^32,33^ and suboptimal performance when paired with annihilators with elevated triplet energies^30^ caused us to search for molecular sensitizers with the capability to sensitize all annihilators used herein.

**Table 1:** Photophysical Properties of the Investigated Annihilators

	*S*_1_^a^ (eV)	*T*_1_^b^ (eV)	Φ_F_^c^
PPD	3.99	2.82^d^	0.88
TP	3.98	2.62^e^	0.92
2PI	3.71	2.22^e^	0.85
PPO	3.67	2.40^e^	0.78
PPF	3.59	2.28	0.79
TIPS-Naph	3.53	2.12^f^	0.77

a First singlet excited state energy, determined from the intersection of normalized absorption and fluorescence spectra.

b First triplet excited state energy, determined from the highest energy peak position of phosphorescence collected at 77 K in MTHF or collected from the literature when applicable.

c Fluorescence quantum yield of optically dilute samples. λ_exc_ = 300 nm, determined relative to TP in deaerated cyclohexane (Φ_F_ = 0.93).^41^

d Reference (42).

e Reference (43).

f Reference (28).

We focused our attention on the recently emerging group of TADF sensitizers^13,37,45−51^ and found that purely organic blue-emitting 2,3,5,6-tetra(9*H*-carbazol-9-yl)benzonitrile (4CzBN), developed by the Zhang group,^52^ was able to sensitize all annihilators efficiently. TADF molecules exhibit small singlet–triplet energy splittings (Δ*E*_S–T_, typically below 0.3 eV), which results from a high degree of intramolecular charge transfer (CT) character in the singlet and triplet excited states. In 4CzBN, this is manifested by the CT absorption band with an onset at around 430 nm (Figure 1B), and the covalent linkage between electron donor and acceptor units further enhances the CT character.^53^

The photophysics of organic TADF compounds has been thoroughly investigated by others,^53−58^ and the key processes of a conventional TADF compound are depicted in the left part of Figure 1C. Upon excitation, the first singlet excited state can decay either nonradiatively or by prompt fluorescence. Because intersystem crossing (ISC) is quite strong in these purely organic molecules, TADF compounds also populate their triplet state efficiently via ISC. Δ*E*_S–T_ then dictates how fast reverse ISC (rISC) proceeds, which together with the rates for nonradiative triplet decay and phosphorescence dictates the lifetime of the triplet state. The recycling of singlet and triplet states ultimately results in TADF from the singlet state, typically on the microsecond timescale. If molecular oxygen (O_2_) is present, the triplet state will be efficiently quenched, and no TADF will be observed.^54^

4CzBN exhibits both prompt fluorescence and TADF in toluene. The fluorescence quantum yield (Φ_F_) and lifetime (τ) of the prompt (PF) component were determined from measurements in air-saturated samples, while the delayed (DF) component was readily observed in oxygen-free samples. The total Φ_F_ of 4CzBN was determined to be 0.64, with Φ_PF_ and Φ_DF_ being 0.11 and 0.53, respectively, well in accordance with previous studies on 4CzBN.^52,58^ τ_PF_ showed minor susceptibility to the presence of oxygen, decreasing from 2.34 ns in oxygen-free solution to 2.22 ns upon exposure to air. The lifetime of the delayed component, τ_DF_, is of particular importance in TTA-UC since it corresponds to the triplet lifetime. τ_DF_ of 4CzBN was determined to be 62 μs, which is sufficiently long to promote diffusion-controlled Dexter-type triplet energy transfer (TET)^60^ upon the addition of an annihilator species. This relatively long lifetime is the result of a rather large Δ*E*_S–T_ of 0.28 eV, thus impeding the rate of rISC. The ISC efficiency can be estimated as 1 – Φ_PF_, yielding Φ_ISC_ = 0.89. The most important photophysical parameters of 4CzBN are summarized in Table 2.

**Table 2:** Photophysical Properties of 4CzBN

	Φ_F_^a^	τ_PF_ (ns)	τ_DF_ (μs)	Φ_ISC_	*T*_1_^b^ (eV)	Δ*E*_S–T_ (eV)
4CzBN	0.11^c^/0.53^d^	2.34^e^/2.22^f^	62.2	0.89	2.71	0.28

a Fluorescence quantum yield of optically dilute samples. λ_exc_ = 405 nm, determined relative to Coumarin 153 in aerated EtOH (Φ_F_ = 0.53).^59^

b First triplet excited state energy, determined from the highest energy peak position of phosphorescence collected at 77 K in MTHF.

c Prompt component.

d Delayed component.

e In oxygen-free solution.

f In air-saturated solution.

### UC Characteristics

To achieve TTA-UC, it is beneficial if the intermolecular TET process between the sensitizer and annihilator outcompetes all intramolecular processes proceeding from the triplet state of the sensitizer. Different versions of the Stern–Volmer equation have previously been used when estimating TET efficiencies from TADF compounds, and examples include using the difference between the quenched and unquenched donor total fluorescence quantum yield^13^ or delayed component lifetime.^49^ Given that the equilibrium between the singlet and triplet state in a TADF compound is perturbed upon the addition of a quencher, the methods mentioned above are riddled with assumptions that are valid only for certain compounds. To ensure that the chosen method was valid for 4CzBN, we performed simulations (Figure S2). The results indicate that probing the changes in τ_DF_ upon quenching of 4CzBN yields excellent agreement with the true TET efficiency, as given by eq S1E. Note that the definition for TET efficiency used herein includes the ISC event, that is, the maximum value for Φ_TET_ = Φ_ISC_ (eq S1E, for a more detailed discussion, see the Supporting Information, Section S2.1).

The quenching behavior was analyzed by titration series with each annihilator species, and the obtained TET rates were calculated using eq S2. The resulting *k*_TET_ are found in Table 3 (see Figure S3 for Stern–Volmer plots). As expected, *k*_TET_ are typically higher for the annihilators with lower-lying triplets (see Table 1 for triplet energies and Figure S4 for the phosphorescence of PPF), but fortunately, endothermic TET from 4CzBN is also possible, yielding *k*_TET_ on the order of 10^8^ M^–1^ s^–1^ to the high-triplet energy annihilator PPD. We note that using phosphorescence spectra of rotationally flexible molecules typically underestimates the triplet energy,^43^ so the energy commonly referenced for TP (2.53 eV)^13,37^ is therefore likely underestimated. We choose instead a value of 2.62 eV, which was obtained from quenching experiments^43^ and which better correlates with the relatively slow TET (*k*_TET_ = 4.1 × 10^8^ M^–1^ s^–1^) observed from 4CzBN to TP.

**Table 3:** Measured Values of Yields and Rates Important in TTA-UC

	Φ_UC__,__g_ | Φ_UC_^a^	*k*_TET_^b^ (×10^9^ M^–1^ s^–1^)	τ_T_^c^ (ms)	*I*_th_^d^ (mW cm^–2^)	*k*_TTA_^e^ (×10^9^ M^–1^ s^–1^)	β_max_^f^	*f*^g^
PPD	0.058 | 0.044	0.18	0.18	4900	2.87	0.67	0.22
TP	0.126 | 0.091	0.41	0.31	1700	3.30	0.77	0.40
2PI	0.044 | 0.039	2.1	0.075	>25,000	0.69	0.27	0.43
PPO	0.140 | 0.124	2.0	1.3	210	1.75	0.91	0.44
PPF	0.130 | 0.102	3.6	0.75	600	1.77	0.85	0.44
TIPS-Naph	0.168 | 0.131	0.80	2.2	220	0.62	0.91	0.54

a UC quantum yield (out of a 0.5 maximum) upon 405 nm cw excitation, determined relative to Coumarin 153 in aerated EtOH (Φ_F_ = 0.53).^59^

b Rate constant for TET from 4CzBN.

c Lifetime of the first triplet excited state.

d Threshold excitation intensity evaluated at β = 0.5.

e Rate constant for TTA.

f Maximum β value as defined by eq 3, estimated at a laser fluence of 18 W cm^–2^.

g Spin-statistical factor, calculated using eq 1 with Φ_TET_ = 0.89 and Φ_TTA_ = β_max_/2.

**Figure 2:**
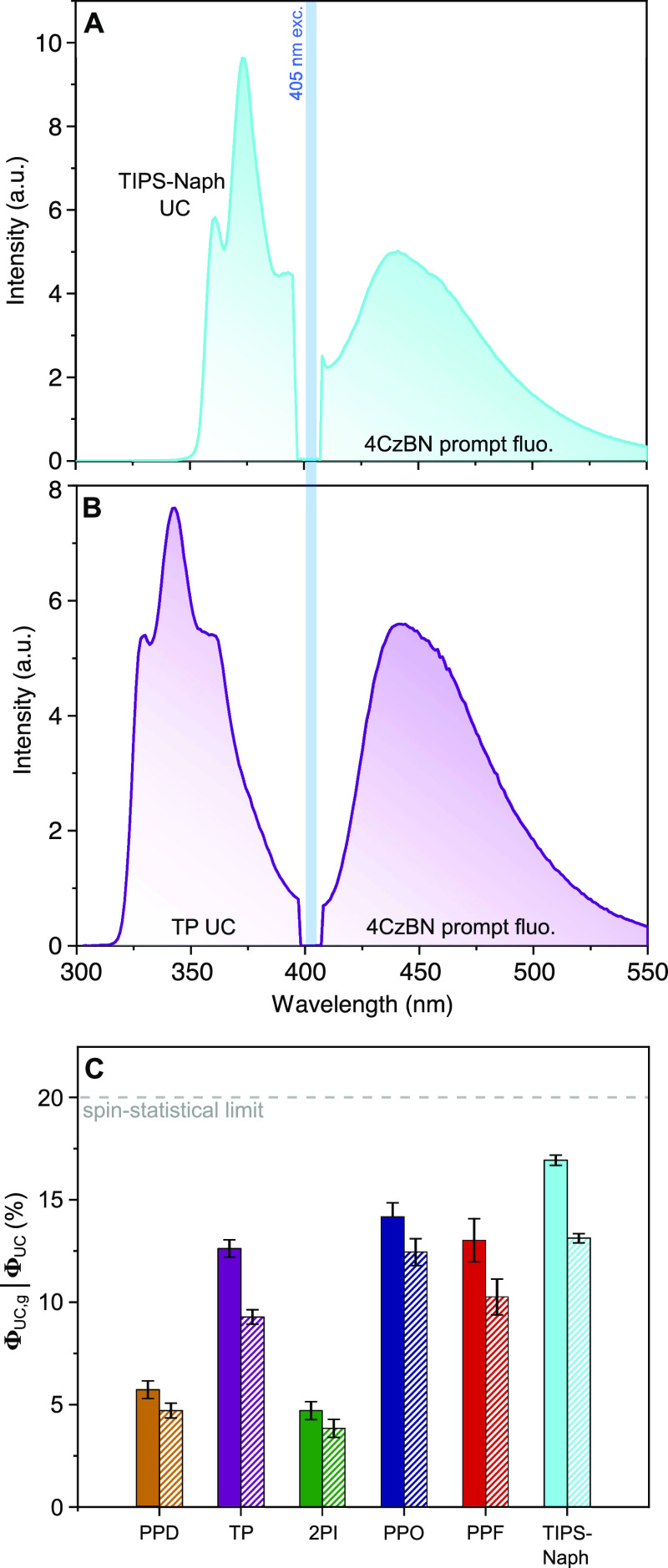
Steady-state emission spectrum of 25 mM 4CzBN and (A) 1 mM TIPS-Naph or (B) 10 mM TP upon 405 nm cw excitation in deoxygenated toluene. The short wavelength feature is UC fluorescence from TIPS-Naph/TP and the low-energy band is residual prompt fluorescence from 4CzBN (with Φ_PF_ ≈ 0.11). (C) Internal (solid) and external (striped) UC quantum yields for each annihilator when paired with 4CzBN, evaluated at a laser fluence of 18 W cm^–2^. Standard deviations based on the results of three independent samples are indicated by black error bars.

With these results at hand, we investigated the TTA-UC performance of the different systems. The concentrations employed for UC measurements were 25 mM of 4CzBN and 10 mM (1 mM for 2PI and TIPS-Naph, *vide infra*) of the annihilator, resulting in systems with endothermic TET (i.e., TET from 4CzBN to PPD) also having Φ_TET_ close to 89% (as calculated by eq S4). Delayed UC fluorescence could be observed from all systems upon 405 nm excitation, and the UC emission spectra of TIPS-Naph and TP are presented in Figure 2A,B. The spectral shapes are marred by the secondary inner filter effect at the high-energy end of the spectrum, which is caused by the overlap of UC emission and sample absorption. This is typically an issue in vis-to-UV UC especially, even though there are examples of sensitizers with limited UV absorption, thus somewhat mitigating this issue.^28,29^ The low-energy band peaking at around 440 nm is residual prompt fluorescence from 4CzBN, which is an inevitable loss-channel in all these systems. Interestingly, this feature can act as an approximate internal quantum yield reference since the prompt component of 4CzBN (with Φ_PF_ = 0.11) should be virtually unaffected by the addition of annihilator species. Unfortunately, sensitizer degradation during measurements (*vide infra*) allows only approximate Φ_UC_ values to be obtained using the prompt component. Coumarin 153 (Φ_F_ = 0.53)^59^ was employed as an external quantum yield reference instead, ensuring high reliability when evaluating Φ_UC_.

When evaluating the annihilators, it is the intrinsic ability to effectively convert low-energy to high-energy light that is of specific interest. The internal, or generated, UC quantum yield (referred to as Φ_UC,g_)^61^ was determined alongside that of the external quantum yield (Φ_UC_). The difference between these mainly lie in that secondary inner filter effects are accounted for when calculating Φ_UC,g_, which affect both the spectral shape and the peak intensities (Figure S5). There is some confusion in the literature regarding the use of Φ_UC_ and Φ_UC,g_, despite recent efforts by in particular Zhou et al. to clarify this issue and standardize the way of reporting, and using, these parameters.^61^ The analysis of intrinsic TTA-UC system parameters is often (erroneously) based on values of Φ_UC_, even though Φ_UC,g_ must be used to determine, for example, the spin-statistical factor. Φ_UC,g_ is often considered as the product of the efficiency of all steps leading up to the emission of UC light (eq 1)
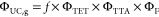
1where *f* is the spin-statistical factor, Φ_TET_ is the TET efficiency (ISC included), Φ_TTA_ is the TTA quantum yield, and Φ_F_ is the annihilator fluorescence quantum yield. Since two low-energy photons are needed to afford one highly energetic singlet, Φ_TTA_ (and subsequentially Φ_UC,g_) has a theoretical maximum of 50%. Reabsorption is accounted for by using the output coupling yield, Φ_out_, with Φ_UC_ = Φ_UC,g_ × Φ_out_.^61^ A lower value for Φ_out_ indicates stronger reabsorption of UC emission by the sample. Another factor that had to be dealt with was that of sensitizer degradation. This is a common issue in vis-to-UV UC systems^36^ and a challenge also faced by the organic light-emitting diode (OLED) community when working with TADF materials in general.^62^ Upon 405 nm continuous wave (cw) excitation, 4CzBN suffered from degradation, which manifested itself both in changes of the absorption spectrum and in loss of fluorescence over time (Figure S6). When paired with an annihilator species, the UC emission intensity typically went down over time, even though efficient TET attenuated the sensitizer degradation (Figure S7).

To determine Φ_UC,g_, a fitting procedure that accounts for reabsorption was employed, and it is explained in detail in Section S2.3 of the Supporting Information. To our delight, all systems investigated yielded relatively high Φ_UC,g_, with the system consisting of 4CzBN/TIPS-Naph in particular yielding a high value of 16.8% (out of a 50% maximum, see Figure 2A for the UC spectrum). This value is to the best of our knowledge the highest vis-to-UV Φ_UC,g_ reported to date and a significant improvement on the previous record.^28−30^ The remaining systems yielded Φ_UC,g_ values ranging from 4 to 14%, and full results are presented in Figure 2C and Table 3. It should be noted that the values achieved for TP (12.6%) and PPD (5.8%), which both emit from singlet states just shy of 4 eV, are multifold improvements on that previously reported^30,63^ and likely result from more efficient TET and, subsequently, more efficient TTA between triplets. The external Φ_UC_ measured for our specific setup yielded Φ_out_ between 0.7 and 0.85, resulting from significant reabsorption of the samples. In TIPS-Naph and PPF, this results from very small Stokes shifts (Figure 1A), causing ground-state annihilators to reabsorb the UC light to a larger extent than in systems with larger Stokes shifts. In PPD and TP, relatively low Φ_out_ instead results from the pronounced absorption feature of 4CzBN between 300–350 nm (Figure 1B), which is part of the spectral region where these annihilators emit. Measurements on all annihilators were also performed in THF, typically yielding lower Φ_UC,g_ and much more pronounced sample degradation (Table S1 and Figure S7).

TIPS-Naph was synthesized in accordance with a literature procedure,^28^ and during experiments, a fluorescent contamination, which has not been reported previously, was discovered. As detailed in Section S2.5 of the Supporting Information, the removal of this contamination by additional cycles of recrystallization lead to a substantial increase in Φ_UC,g_. This could potentially explain why we see a higher external Φ_UC_ (13.1%) than that in other studies using TIPS-Naph (Φ_UC_ ≈ 10%), in which Φ_TET_ is reported to be close to unity.^28,29^

Our group has previously investigated the locked t-stilbene compound 5,10-dihydroindeno[2,1-*a*]indene (I2),^30^ a highly fluorescent compound that unfortunately suffers from very low solubility in toluene. 2PI was chosen as a potentially more soluble equivalent to I2, and the solubility was indeed much higher. When samples containing 10 mM 2PI were used for TTA-UC, however, some light scattering was evident in the absorption. Additionally, the UC signal increased strongly over time during 405 nm excitation, reaching a maximum value after approximately 30 min (Figure S8A). The measured Φ_UC,g_ for 2PI was low (1.0%), which is a lower estimate given that the extended laser exposure not only causes the UC emission signal to increase but also the sensitizer to degrade. Upon lowering the 2PI concentration to 1 mM, the scattering decreased significantly, indicating that the observed behavior was due to 2PI not being fully solvated at 10 mM. At 1 mM, Φ_UC,g_ went up to 4.4%, and no signal increase was observed over time (Figure S8B).

### Probing Triplet Kinetics Using Time-Resolved Emission

To understand the differences in Φ_UC,g_ between the annihilators, we examined the kinetics of the UC samples. There are several important rate constants and parameters needed to properly evaluate TTA-UC systems, for example, the annihilator triplet excited state lifetime, the TTA rate constant (*k*_TTA_), and the excitation threshold intensity (*I*_th_). In the following section, we show that these can be determined from the same series of time-resolved UC emission measurements, thus circumventing the need for more challenging transient absorption measurements altogether.

A key factor dictating TTA-UC performance in solution is the annihilator triplet lifetime (τ_T_). A long τ_T_ is needed to allow annihilator triplets to diffuse and encounter, resulting in the creation of emissive singlet states via TTA. τ_T_ was measured using a previously developed method^23,64^ where the excitation intensity (*I*_EX_) dependence on the UC emission kinetics is used (eq 2).
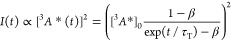
2Here, *I*(*t*) is the time-dependent UC emission intensity, [^3^A*] is the annihilator triplet concentration, *t* is the time, and β is a dimensionless parameter indicating the fraction of triplets that initially decay by second-order channels, as defined by eq 3. In other words, β represents a system’s TTA efficiency (with a possible maximum of 100%), and Φ_TTA_ may be calculated as β/2 given that these are evaluated at identical experimental conditions.
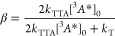
3Here, *k*_T_ (=1/τ_T_) is the intrinsic first-order rate constant of annihilator triplet decay and [^3^A*]_0_ is the initial annihilator triplet concentration. Lowering *I*_EX_ impedes triplet formation and subsequent TTA, thus lowering β. *k*_TTA_ could be extracted from eq 3 if [^3^A*]_0_ could be estimated. The rate constants used above also relate to the *I*_th_ (eq 4), which represents where the steady-state UC emission makes a transition from a quadratic dependence on *I*_EX_, indicating that the intrinsic decay of annihilator triplets dominate, to a linear dependence on *I*_EX_, indicating that TTA instead dominates.^11,65^
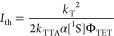
4Here, α and [^1^S] are the absorption cross-section and ground-state concentration of the sensitizer, respectively.

Our group has previously determined *k*_TTA_ for compounds based on 9,10-diphenylanthracene (DPA) using a method where both time-resolved emission and transient absorption measurements are needed.^23,66,67^ While the same method in principle is applicable to any system, the spectral overlap between the prompt fluorescence of 4CzBN and the T_1_ → T_*n*_ absorption of, for example, PPO^30^ complicates matters considerably for the systems used here. A new method has instead been developed, which relies solely on time-resolved emission measurements of the UC samples, thus circumventing the need for transient absorption.

**Figure 3:**
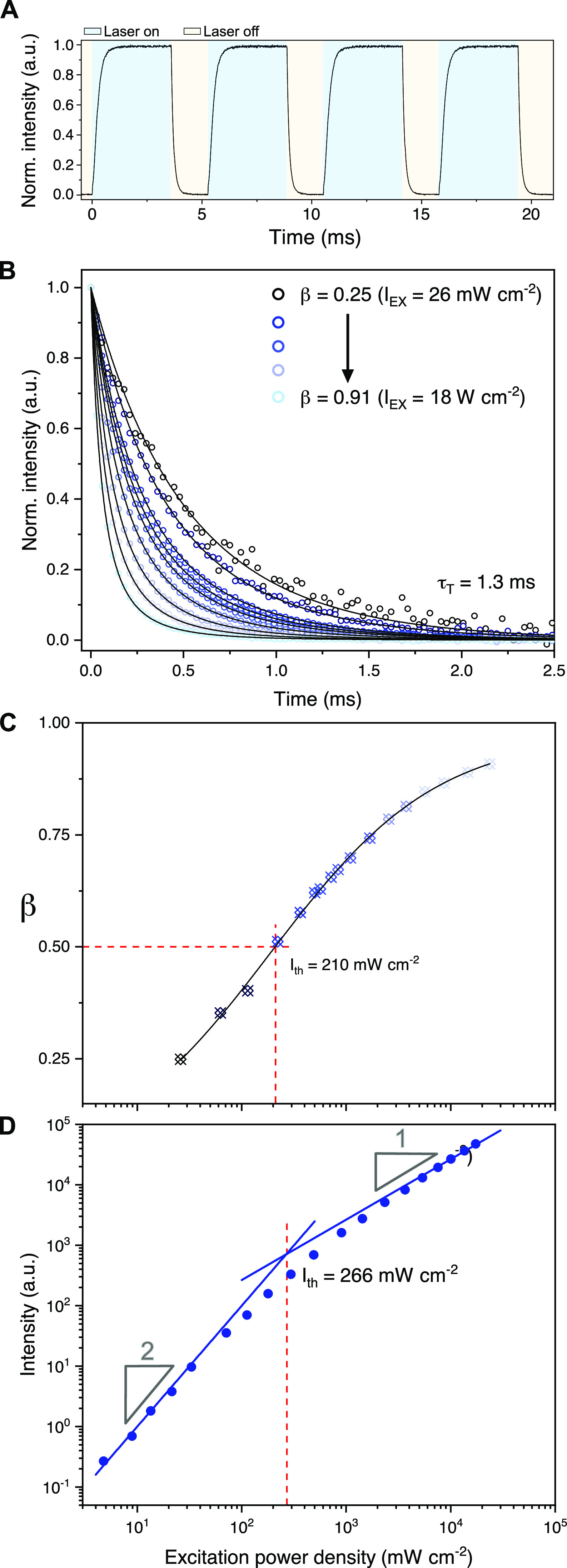
(A) Example of kinetic traces of the UC emission. The population time (laser on) was chosen such that the UC intensity plateaued before decaying (laser off). (B) Normalized excitation intensity-dependent kinetics of the UC emission of PPO (i.e., the decay during the “laser off” state from panel A). Some measurements are omitted for clarity purposes. (C) Evaluation of threshold excitation intensity (*I*_th_) at β = 0.5 for PPO. The solid line is included as a guide to the eye. (D) Conventional method of determining *I*_th_ for PPO.

Instead of using a nanosecond pulsed laser for excitation, we used a 405 nm modulated cw laser diode, which we coupled to a pulse generator. This way, we could control the exact length of the excitation pulse such that the sample emission had reached a quasi-steady-state before the excitation light was turned off and the UC emission started to decay (Figure 3A). This means that [^3^A*]_0_ can be estimated to be equal to the steady-state triplet concentration ([^3^*A*_SS_]) at a given *I*_EX_. The steady-state rate expression for [^3^*A*_SS_] is given by eq 5
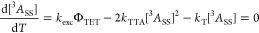
5

The excitation rate, *k*_exc_, is easily estimated from the sample absorbance at the excitation wavelength and the excitation power (eq S8). Setting [^3^A*]_0_ = [^3^*A*_SS_], a simple and solvable equation system with only two unknowns, *k*_TTA_ and [^3^*A*_SS_], is obtained from eqs 3 and 5.
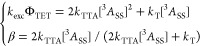
6

Consequently, it is possible to estimate *k*_TTA_ using the exact same measurements that were used to determine τ_T_ (=1/*k*_T_) of the annihilators. An additional benefit is that it is now possible to directly relate *I*_th_ and β. Since β is evaluated at [^3^*A*_SS_], *I*_th_ is the excitation intensity that yields β = 0.5.^68^*I*_th_ may, thus, be estimated from only a few measurements of the UC emission decay, in which the excitation intensity is varied to yield values of β slightly above and below 0.5.

Measurements of the UC decay kinetics at different *I*_EX_ values were performed, followed by a global fitting procedure in which τ_T_ was fitted to a global constant value while allowing β to vary (Figure 3B, see Supporting Information Section S2.4 for more details). The results show that PPO has a lifetime of 1.3 ms, which is substantially longer than that previously reported for UC systems employing PPO^14,30,34^ but close to that obtained from flash photolysis experiments.^69^ The longest lifetime was found for TIPS-Naph at 2.2 ms, resonating well with its impressive performance in terms of Φ_UC,g_. The remaining lifetimes span from 0.075 to 0.75 ms (Table 3), which are much shorter than those often found in visible emitters based on anthracene, where lifetimes on the order of several milliseconds are common.^23,67^ At high *I*_EX_, most annihilators still show β values relatively close to unity, indicating that the TTA pathway dominates at high *I*_EX_ (Figure S9).

To utilize TTA-UC with sunlight, it is beneficial if the system works efficiently at the solar flux at Earth’s surface, which is only a few milliwatts per squared centimeter in the wavelength region of interest here. For this purpose, all investigated systems show unsatisfactory high *I*_th_, with values above 200 mW cm^–2^ (Figures 3C,D and S10). This emanates from the fact that τ_T_ is quite short in these annihilators, combined with a relatively low molar absorptivity of 4CzBN at the excitation wavelength of 405 nm (ε ≈ 7000 M^–1^ cm^–1^). Comparison between *I*_th_ obtained by evaluation at β = 0.5 (Figure 3C) and the traditional evaluation of *I*_th_ obtained from fitting the steady-state intensity to slopes 1 and 2 (Figure 3D) yields good agreement between the methods.

The *k*_TTA_ rates were determined from the same measurements as those detailed in the Supporting Information, and the obtained rates are presented in Table 3. Interestingly, TIPS-Naph shows the lowest *k*_TTA_ of the annihilators investigated here (6.2 × 10^8^ M^–1^ s^–1^); PPO and PPF show similar rates of around 1.75 × 10^9^ M^–1^ s^–1^; while, for example, TP has an almost 2 times higher rate constant of 3.3 × 10^9^ M^–1^ s^–1^. We note that the measured value of *k*_TTA_ for PPO is approximately 3 times lower than that reported previously.^14^ These results indicate that while the rate of the TTA event itself obviously affects the UC efficiency, it is the annihilator triplet lifetime that preferentially dictates the outcome. This is hardly surprising but worth reiterating, and great care should be given when evaluating especially the triplet lifetime of the annihilator.

## Discussion

### TADF Sensitizers: Drawbacks and Opportunities

As is evident from this study, using TADF compounds as the sensitizers in TTA-UC holds great promise. The most obvious advantage compared to other sensitizers yielding decent Φ_UC_ in vis-to-UV TTA-UC (i.e., Ir complexes and quantum dots containing heavy metals such as Cd and Pb)^28,30^ is that TADF compounds are purely organic, consisting only of earth-abundant, nontoxic elements. They are, thus, well-suited for future large-scale operation, which is not the case for Ir complexes, despite possessing promising photophysical properties otherwise. Additionally, due to the OLED community’s increasing interest in TADF compounds during the last decade, there is a huge variety of available molecules with different energy levels and triplet excited state lifetimes, of which the latter in many cases are orders of magnitude longer than those found in, for example, Ir complexes.^70^

**Figure 4:**
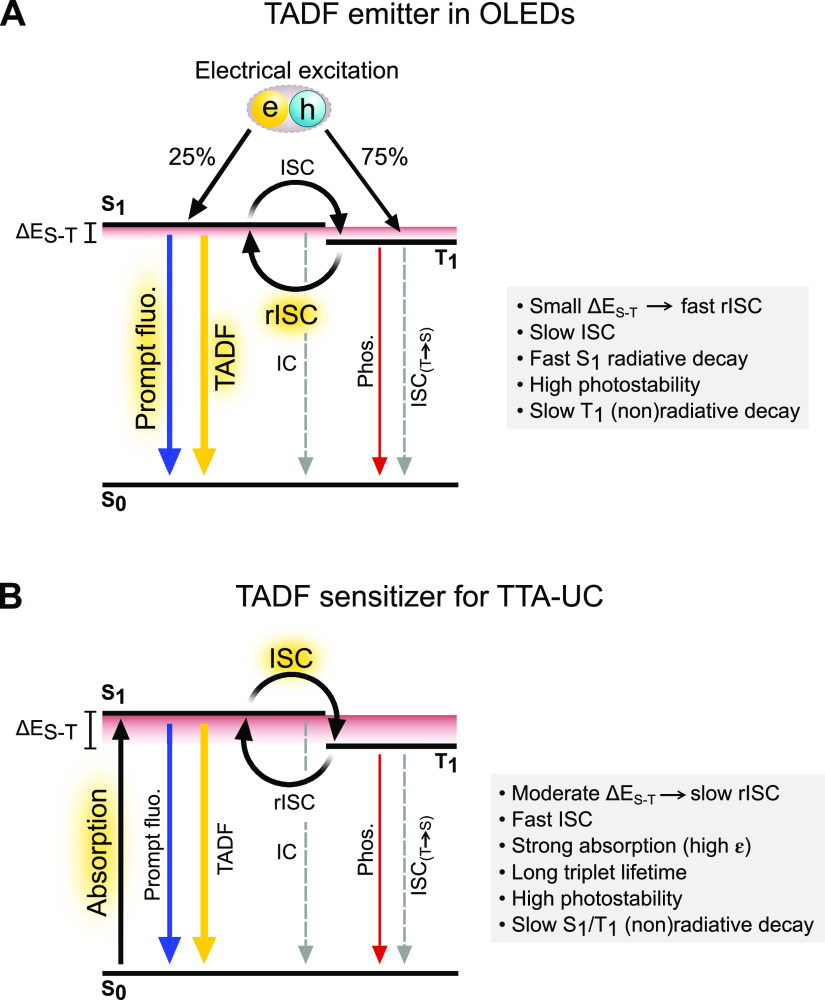
Comparison between the functionality and properties of TADF compounds as (A) emitters in OLED devices and (B) sensitizers in TTA-UC. Processes that should be promoted in the respective settings are highlighted.

Making the best use of existing TADF compounds in TTA-UC schemes is, however, not straight-forward. The sought-after qualities for use in OLEDs differ significantly from what is needed in a typical sensitizer, meaning that current TADF design in many cases has gravitated toward compounds not suitable for TTA-UC. One crucial benefit in both contexts is the access to small singlet-triplet energy splittings (Δ*E*_S–T_). In OLEDs, the excited states are created by means of electricity, and the resulting distribution is dictated by spin statistics, leading to 75% triplets and 25% singlets (Figure 4A).^55^ Highly efficient rISC to generate a higher fraction of emissive singlets is, thus, one of the most important properties of TADF compounds in the context of OLEDs and is a process that is sped up in molecules with small Δ*E*_S–T_ (generally, *k*_rISC_ ∝ exp[−Δ*E*_S–T_/*k*_B_*T*]).^53^ In TTA-UC, a small Δ*E*_S–T_ enables larger apparent anti-Stokes shifts since the initial energy loss during the ISC event is smaller than that in typical sensitizers containing heavy metals.^13^ Once the triplet has been populated, it is instead beneficial if rISC is inefficient since the generated exciton should be transferred to the annihilator instead of returning to the singlet manifold. A too small Δ*E*_S–T_ might therefore inhibit efficient TET even if the annihilator concentration is kept high.^13,38^ An intermediate Δ*E*_S–T_ (0.1 eV < Δ*E*_S–T_ < 0.2 eV), enabling relatively large apparent anti-Stokes shifts and slow rISC simultaneously, should be favored. Even smaller Δ*E*_S–T_ could potentially be used by invoking strategies in which the rISC process is slowed down by clever molecular design.^71^ The TET event is further limited by the amount of prompt fluorescence in systems with TADF-type sensitizers. Contrary to what is wanted for OLED applications, the prompt fluorescence quantum yield should be as low as possible in TTA-UC settings to promote efficient TET (Figure 4B).

Recently, some progress in this area has been made. Wei et al. reported two new multiresonance TADF sensitizers, which when paired with TIPS-Naph or a derivative thereof afforded green-to-UV TTA-UC for the first time.^45^ While a relatively modest Φ_UC_ value of 3.8% is reported, they managed to reach a low *I*_th_ of 9.2 mW cm^–2^. Part of the success is ascribed to the high molar extinction that was determined for these sensitizers (ε > 10^5^ M^–1^ cm^–1^), enabled by limiting their structural flexibility and by including electron-deficient boron covalently bonded to the donor units.

4CzBN possess several of the sought-after properties of a sensitizer, with weak prompt fluorescence, a long-lived delayed component, and slow rISC (Figure 4B). Its major drawback is the (for most systems) unnecessarily high singlet and triplet energies, which forbid excitation at wavelengths >430 nm, leading to significant energy loss during ISC and TET. Additionally, some photo instability of the UC samples was detected, which was ascribed to the degradation of 4CzBN, an issue that can be alleviated by the addition of bulky substituents.^52^ Finding complementary compounds with similar characteristics to 4CzBN but with lower excited state energies will be needed to further improve green-to-UV UC, which is especially interesting for solar applications given the vast amounts of green light in the solar spectrum.

### Considerations on Annihilator Design

Novel TADF sensitizers can contribute to the improvement of vis-to-UV TTA-UC systems, but what is perhaps even more crucial is the pursuit of new annihilators.^28,38,45^ Design principles that hold true for annihilators in general must obviously be upheld, such as high Φ_F_ and a long τ_T_, but for UV-emitting systems, additional considerations should be taken into account. As touched upon previously, many vis-to-UV TTA-UC systems suffer from low photostability, which follows from the relatively high energy of the states involved. This aspect has recently been investigated in greater detail by Murakami et al., gaining important insights into how the energy levels of the sensitizer and annihilator affect the photostability of TTA-UC systems in solution.^36^ They observed a correlation between the main degradation pathway and the energy difference between the LUMO levels of the annihilator and solvent. In our study, we found no evidence of annihilator degradation during UC experiments, and we primarily ascribe the slight decrease in UC emission over time to sensitizer degradation.

Another aspect that is especially relevant for vis-to-UV TTA-UC is the exaggerated thermodynamic driving force for TTA typically found in UV-emitting species. This is the case for the compounds investigated herein: [2 × *E*(T_1_) – *E*(S_1_)] ≥ 0.7 eV for all species, with PPD in particular having a driving force of almost 1.6 eV. If this substantial energy loss could be mitigated, substantially larger apparent anti-Stokes shifts could be realized. The relative lowering of the triplet energies should perhaps be the primary focus as this would enable excitation at longer wavelengths than those currently possible. A few studies have investigated substituent effects on the energetic landscape of polyacene emitters,^67,72^ with the study by Fallon et al. specifically showing that the first excited singlet state can be lowered by adding TIPS substituents while keeping the first triplet excited state relatively constant.^72^ Such modifications may be useful for other spectral ranges, but the effect is the opposite of what is required to improve vis-to-UV TTA-UC. A recent study by Zähringer et al. reported a new annihilator with a highly energetic S_1_ state at 4.04 eV.^38^ Surprisingly, this compound also exhibits a relatively low-lying T_1_ state calculated to lie at 2.48 eV, enabling excitation with 447 nm light. It was not detailed by the authors why T_1_ had such a low energy, but their results indicate that substituent effects still could be of interest when modulating excited-state energies for vis-to-UV TTA-UC.

Controlling not only the energy of T_1_ but also that of T_2_ is of significance. In molecules, such as perylene and rubrene, in which the spin-statistical factor *f* has been determined to lie above the commonly encountered value of 2/5,^40,73^ with the energy difference [2 × *E*(T_1_) – *E*(T_2_)] < 0. In perylene, this difference is strongly negative, efficiently shutting down the creation of the T_2_ state upon TTA, causing *f* to approach unity.^40^ In rubrene, however, *f* is reported to lie around 0.6 in solution,^73^ and the creation of T_2_ during TTA is only slightly endothermic.^39^ A recent study by Bossanyi et al. verifies that T_2_ is formed during TTA in rubrene but that the energy alignment between T_2_ and S_1_ allows fast high-level rISC (HL-rISC) from T_2_ to S_1_ to occur, outcompeting nonradiative decay from T_2_ to T_1_.^39^ HL-rISC has also been found in anthracene derivatives (not DPA however)^74^ and should be considered as a potential avenue to increase *f* beyond 2/5. This pathway is very sensitive to the precise alignment of S_1_, T_1_, and T_2_ energies, and the study by Bossanyi et al. suggests that in cases where [2 × *E*(T_1_) – *E*(T_2_)] approaches zero, *f* may in fact approach unity in molecules where HL-rISC occurs. Finally, from simulations, the same study states that intermolecular geometry can affect *f*, with parallel geometries giving rise to higher values. For the annihilators used herein, [2 × *E*(T_1_) – *E*(T_2_)] is expected to be much greater than zero. Additionally, S_1_ is expected to lie several hundreds of millielectron volts above T_2_ for most annihilators (Table S2), suggesting that HL-rISC is inefficient in these molecules. Most of the investigated annihilators show an expected value of approximately 0.4, but our results also indicate that *f* takes a larger value than 2/5 in TIPS-Naph (0.54) but a lower value for PPD (0.22, Table 3). While the reason for this is unclear, we note that the calculated S_1_ energy of TIPS-Naph lie approximately 100 meV below that of T_2_ (Table S2). This energy alignment could potentially enable exothermic HL-rISC from T_2_ to S_1_ in TIPS-Naph, which would explain the higher *f* value. Regardless, it is obvious that the spin-statistical factor is still not fully understood and that efforts to elucidate the true nature of it will be needed to predict and design efficient annihilators.

## Conclusions

In this work, we show that the internal UC quantum yield of visible-to-UV TTA-UC systems may approach the often-encountered spin-statistical limit of 20%. We do so by pairing six different annihilators with the purely organic, high triplet energy sensitizer 4CzBN that exhibits efficient ISC and a long triplet lifetime. The results show that the TTA-UC pair 4CzBN/TIPS-Naph achieve a record-setting 16.8% internal UC quantum yield (out of a 50% maximum), and high internal quantum yields are reached when using PPO (14.0%), PPF (13.0%), or TP (12.6%) as annihilators as well. We also show that the same set of time-resolved emission measurements can be used to determine the annihilator triplet lifetime, the rate constant of TTA, and the threshold excitation intensity, all of which are important parameters to probe when evaluating TTA-UC systems. The importance of having long-lived annihilator triplets is reinforced as our results show that both the TTA-UC quantum yield and the threshold excitation intensity benefits from this. Using 4CzBN as the sensitizer limits the achievable anti-Stokes shifts, and our results are discussed in the context of extending the excitation wavelength further into the visible region. The development of high-efficiency vis-to-UV TTA-UC systems will require both new sensitizer and annihilator compounds, and finding avenues to control and alter the singlet and triplet energy levels of these will be crucial in order to combine high efficiencies with, for example, excitation with green light.

## Supporting Information Available

Detailed description of experimental setups, additional spectroscopic and modeling data, calculation details, synthesis details, and proton and carbon NMR spectra (PDF)

## Footnotes

a In the literature, both external and internal UC quantum yields are reported, of which the latter compensates for secondary inner-filter effects. The difference between these is further discussed in the Results section.
