# Supplementary material for: Approaching the Spin-Statistical Limit in Visible-to-Ultraviolet Photon Upconversion
Source: J Am Chem Soc. 2022 Feb 17;144(8):3706–16. doi: 10.1021/jacs.1c13222 (PMC8895402; doi:10.1021/jacs.1c13222)
Supplement: Supplementary file 1 — ja1c13222_si_001.pdf [file ja1c13222_si_001.pdf]

Supporting Information

Approaching the Spin-Statistical Limit in Visible-to-Ultraviolet Photon Upconversion

Axel Olesund[1], Jessica Johnsson[1], Fredrik Edhborg[1], Shima Ghasemi[1], Kasper Moth-Poulsen[1,2,3], and Bo Albinsson[1,*]

[1]Department of Chemistry and Chemical Engineering, Chalmers University of Technology, 412 96 Gothenburg, Sweden

[2]Institute of Materials Science of Barcelona, ICMAB-CSIC, 08193 Bellaterra, Barcelona, Spain

[3]Catalan Institution for Research and Advanced Studies ICREA, Pg. Lluís Companys 23, Barcelona, Spain

*E-mail: balb@chalmers.se

1. CHEMICALS AND EXPERIMENTAL METHODS ---------- 2
1.1 CHEMICALS ---------- 2
1.2 INSTRUMENTATION AND OPTICAL MEASUREMENTS ---------- 2
2. PHOTOPHYSICAL CHARACTERIZATION ---------- 3
2.1 DETERMINATION OF TET RATES FROM TADF COMPOUNDS ---------- 3
2.2 TRIPLET ENERGIES OF ANNIHILATOR COMPOUNDS ---------- 6
2.3 STEADY-STATE UPCONVERSION ---------- 7
2.4 DETERMINATION OF K_T, K_{TTA}, AND I_{TH} USING TIME-RESOLVED EMISSION ---------- 11
2.5 TIPS-NAPH CONTAMINATION ---------- 14
3. DENSITY FUNCTIONAL THEORY CALCULATIONS ---------- 15
4. SYNTHESIS OF TIPS-NAPH ---------- 15
5. REFERENCES ---------- 18

1. Chemicals and experimental methods

1.1 Chemicals

1,4-dibromonaphthalene, bis(triphenylphosphine)palladium(II) dichloride ($Pd(PPh_3)_2Cl_2$), CuI, PPh_3, diisopropylamine, triisopropylsilylacetylene, 2-phenylindene (2PI), and toluene (spectroscopic grade) were purchased from Sigma-Aldrich. 2,5-diphenyloxazole (PPO), 2,5-diphenyl-1,3,4-oxadiazole (PPD), and *p*-terphenyl (TP) were purchased from Radiant Dyes. 2,5-diphenylfuran (PPF) was purchased from Tokyo Chemical Instruments (TCI). 2,3,5,6-tetra(9H-carbazol-9-yl)benzonitrile (4CzBN) was purchased from Ossila.

1,4-bis((triisopropylsilyl)ethynyl)naphthalene (TIPS-Naph) was synthesized according to a literature procedure (*vide infra*).[1] THF used for the synthesis of TIPS-Naph was dried on an MBraun MB SPS-800 solvent purification system. Column chromatography was performed on a Biotage Isolera using pre-packed silica columns (50g Biotage® SNAP Cartridge). ^{1}H-NMR and ^{13}C-NMR spectra were obtained at 400 MHz.

1.2 Instrumentation and optical measurements

Steady-state absorption spectra were recorded on a Varian-Cary 50 Bio UV−vis spectrophotometer and steady-state fluorescence measurements were carried out on a Spex Fluorolog 3 spectrofluorometer (Horiba Jobin Yvon). The prompt fluorescence lifetimes of 4CzBN were measured using a time correlated single photon counting (TCSPC) setup using PicoQuant laser diodes (405 nm) and a PMT detector (10 000 counts, 4096 channels). Steady-state upconversion fluorescence measurements were performed on a home-built system using a continuous-wave 405 nm OBIS laser (Coherent) as the excitation source. The measured maximum power output was 87.3 mW and the laser beam diameter was 0.8 mm. A linear variable neutral density (ND) filter was used to vary the laser intensity, and data were recorded using home-built LabView software.

Nanosecond time-resolved emission measurements were performed on a home-built system using a continuous-wave 405 nm OBIS laser (Coherent) coupled to a pulse generator as the excitation source. A Cornerstone 130 monochromator (Oriel Instruments) was used when measuring the transient signals at 440 nm from 4CzBN. For transient measurements of upconverted light, a 300-390 nm band-pass filter was instead used after the sample to maximize the signal intensity. The signals were collected with a 9-stage PMT coupled to a Tektronix TDS 2022 oscilloscope. The optical response time of the PMT was set to be much shorter than the measured decay. All photophysical measurements were carried out in toluene using 2 mm quartz

cuvettes. All samples were prepared in a nitrogen glovebox (Innovative Technologies) with <0.7 ppm oxygen levels and sealed with air-tight cap screws and parafilm. Temperature-dependent measurements were performed using a liquid nitrogen cryostat (Oxford Instruments) connected to a temperature controller.

Fluorescence quantum yields of the annihilators were determined using relative actinometry, utilizing deoxygenated *p*-terphenyl in cyclohexane ($\Phi_F = 0.93$)[2] as the reference compound.

2. Photophysical characterization

2.1 Determination of TET rates from TADF compounds

When discussing triplet energy transfer (TET) one usually considers only the actual TET event when evaluating the TET efficiency (Φ_{TET}), i.e. the ratio between produced donor and quencher triplets. In TADF compounds the excited state equilibrium is perturbed upon addition of the quencher, and Φ_{TET} will depend also on the ISC/rISC events, as shown below.

The steady-state rate equations for the first excited singlet and triplet states in the presence of a quencher (i.e. annihilator) species are given by Equations S1A and S1B, respectively.

$$\frac{d[S_1]}{dt} = k_{exc} - (k_F + k_{nr,S} + k_{ISC})[S_1] + k_{rISC}[T_1] = 0 \quad \text{(S1A)}$$

$$\frac{d[T_1]}{dt} = k_{ISC}[S_1] - (k_{ph} + k_{nr,T} + k_{rISC} + k_{TET}[Q])[T_1] = 0 \quad \text{(S1B)}$$

Φ_{TET} is defined by Equation S1C:

$$\Phi_{TET} = \frac{k_{TET}[Q][T_1]}{k_{exc}} = \{replace\ k_{exc}\ with\ the\ equality\ from\ Equation\ S1A\} = \frac{k_{TET}[Q][T_1]}{(k_F+k_{nr,S}+k_{ISC})[S_1] - k_{rISC}[T_1]} \quad \text{(S1C)}$$

$[S_1]$ may be derived from Equation S1B as expressed in Equation S1D.

$$[S_1] = \frac{(k_{nr,T} + k_{ph} + k_{rISC} + k_{TET}[Q])}{k_{ISC}}[T_1] \quad \text{(S1D)}$$

Combining Equations S1C and S1D yields the final expression for Φ_{TET}, Equation S1E.

$$\Phi_{TET} = \frac{k_{ISC}k_{TET}[Q]}{(k_{rISC}+k_T)(k_F+k_{nr,S}+k_{ISC}) - k_{ISC}k_{rISC}} \quad \text{(S1E)}$$

Here, $k_F/k_{nr,S}$ are the rate constants for radiative/non-radiative decay from the first singlet excited state, k_{ISC}/k_{rISC} are the rate constants for intersystem crossing (ISC)/reverse ISC, and $k_T = k_{ph} + k_{nr,T} + k_{TET}[Q]$ is the sum of all decay processes from the first triplet excited state, T_1 ($k_{ph}/k_{nr,T}$ are the rate constants for radiative/non-radiative decay from T_1, k_{TET} is the rate constant for TET, and [Q] is the quencher concentration). Using this definition the maximum Φ_{TET} is equal to Φ_{ISC}. All rates are illustrated in Figure S1. To evaluate this expression all these rate constants must, thus, be determined. This is possible to do but requires a lot of work, especially if the ratio $\Phi_{DF}/\Phi_{PF} < 4$ in the TADF compound of interest.[3]

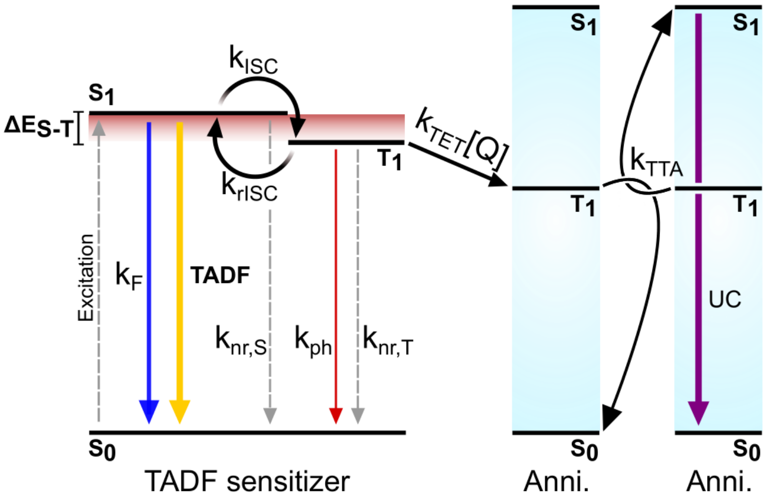

Figure S1. Jablonski diagram illustrating the rate constants relevant for TTA-UC with a TADF-type sensitizer.

In sensitizers which exhibit room-temperature phosphorescence, such as iridium complexes and metal porphyrins, the Stern-Volmer equation (Equation S2) is typically used when determining k_{TET}.

$$\frac{F_0}{F} = \frac{\tau_0}{\tau} = 1 + k_{TET}\tau_0[Q] \quad \text{(S2)}$$

Here, F/F_0 and τ/τ_0 are the quenched/unquenched donor emission (typically phosphorescence) intensities and lifetimes, respectively. In TADF compounds the excited state equilibrium is perturbed upon addition of a quenching species, making the above expression incorrect. Versions in which the phosphorescence emission is replaced by the total fluorescence emission has been used instead,[4] while in some other cases the change in lifetime of the delayed

component (τ_{DF}) upon quenching has been used as a proxy.[5] To explore the validity of each of these approximate methods, simulations were performed. The results are presented in Figure S2. For the simulations the following assumed rates were used: $k_{ISC} = 5 \times 10^8$ s^{-1}, $k_F = 7 \times 10^7$ s^{-1}, $k_{nr,S} = 0$, $k_{ph} + k_{nr,T} = 2 \times 10^4$ s^{-1}. k_{rISC} was calculated using Equation S3 for each value of $\Delta E_{S\text{-}T}$.

$$k_{rISC} = k_{ISC} e^{-\Delta E_{S-T}/RT} \tag{S3}$$

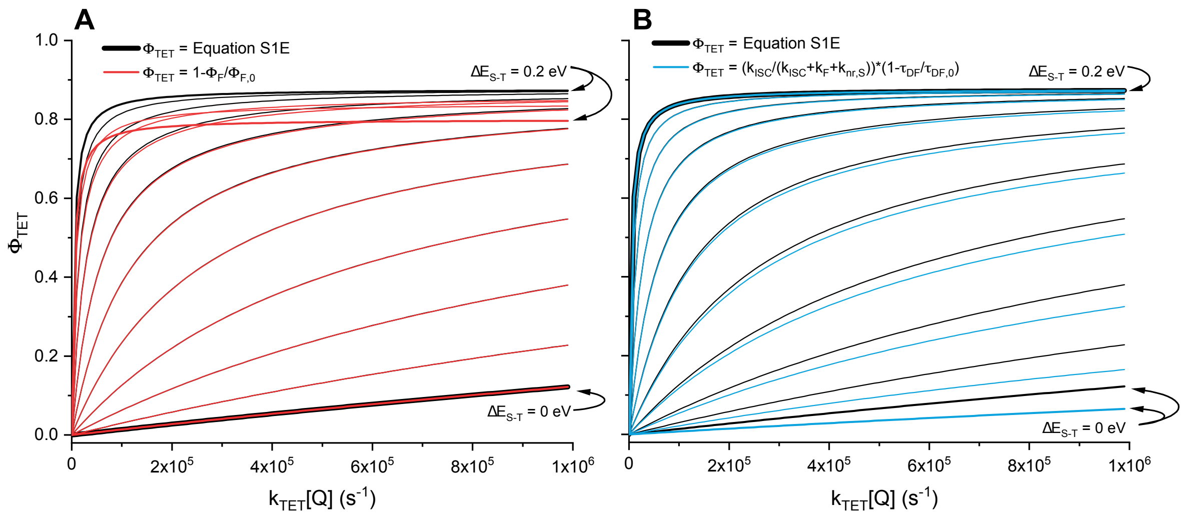

Figure S2. Simulated TET quantum yields as a function of quencher concentration using different expressions. (A) Comparison between Φ_{TET} according to Eq. S1E (black) and according to a Stern-Volmer type expression based on the total (prompt + delayed) fluorescence (red). (B) Comparison between Φ_{TET} according to Eq. S1E (black) and according to a Stern-Volmer type expression based on the lifetime of the delayed component (blue).

While the two different methods are susceptible to the exact values of different rate constants, some qualitative conclusions may be drawn. In general, using the steady-state fluorescence intensity is a good approximation for TADF compounds with small $\Delta E_{S\text{-}T}$ (<0.1 eV, Figure S2A), while using the lifetime of the delayed component as the proxy is more accurate for compounds with larger $\Delta E_{S\text{-}T}$ (>0.1 eV, Figure S2B). For 4CzBN, which has a quite large $\Delta E_{S\text{-}T}$ of 0.28 eV, using only the lifetime of the delayed component as the proxy is likely to yield a very good estimate of Φ_{TET}. One should however always be careful before employing either method and make sure that the implied assumptions are valid for the particular system.

The results from the quenching experiments based on the difference in τ_{DF} of 4CzBN upon titration with respective annihilator is presented in Figure S3 and Table 2 of the main text. The TET efficiency was calculated using Equation S4, where $k_T = k_{ph} + k_{nr,T} = 1/\tau_T$.

$$\Phi_{TET} = \Phi_{ISC} \frac{k_{TET}[Q]}{k_{TET}[Q] + k_T} \tag{S4}$$

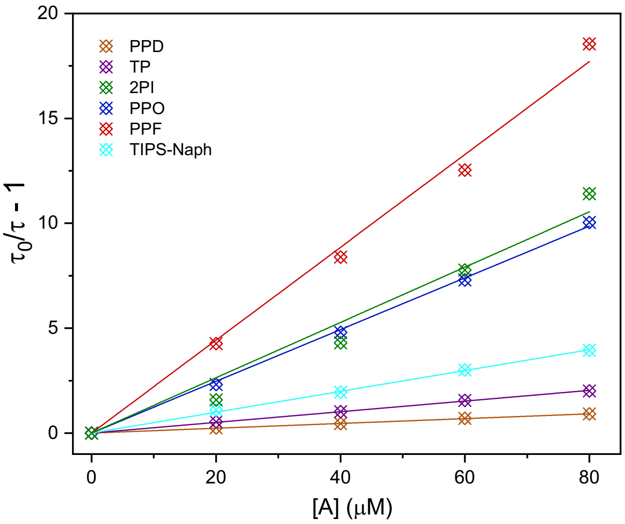

Figure S3. Stern-Volmer plots for the quenching of 4CzBN upon addition of different annihilators.

2.2 Triplet energies of annihilator compounds

When possible the T_1 energies were obtained from the literature (see Table 1 in the main text). No values were found for PPF, and its T_1 energy was estimated from the peak position of its phosphorescence measured in distilled 2-methyltetrahydrofuran (Figure S4). This peak is at 544 nm, yielding a T_1 energy of 2.28 eV.

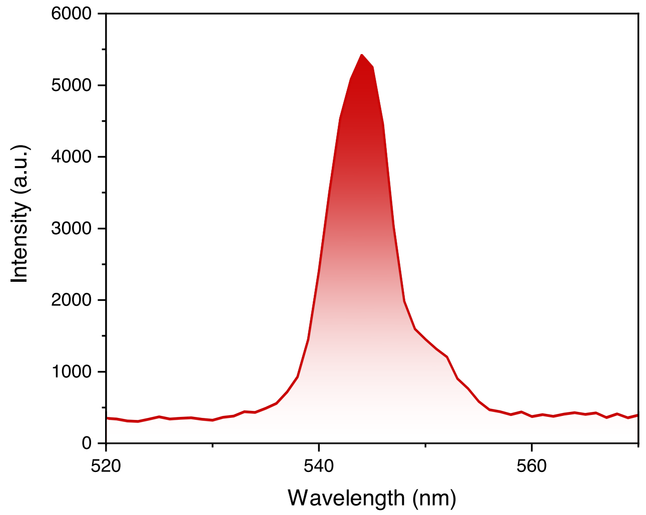

Figure S4. Phosphorescence spectrum (λ_{exc} = 348 nm) of PPF in 2-methyltetrahydrofuran.

2.3 Steady-state upconversion

To evaluate the annihilator performance both the internal, or generated, upconversion quantum yield ($\Phi_{UC,g}$)[6] and the external quantum yield (Φ_{UC}) were determined. $\Phi_{UC,g}$ is of most interest when investigating the intrinsic properties of the annihilators and accounts for reabsorption effects, whereas Φ_{UC} is a sample-dependent metric which depicts the number of photons actually detected from each UC sample.

To determine $\Phi_{UC,g}$ we used a procedure reminiscent of that previously deployed in our group.[7] In all UC measurements 2 mm cuvettes were used and the shorter path length was directed towards the detection source to minimize reabsorption. After the UC spectrum had been collected, the emission spectrum of an optically dilute annihilator sample was fitted to match the spectral region where the UC sample shows low absorption (Figure S5, blue spectra). The fitting was typically performed at 365 nm. The final emission spectrum was integrated (F_{UC}) and compared to the integrated emission of the reference sample Coumarin 153 in air-saturated EtOH (Φ_r = 0.53).[8] Equation S5 was then used to calculate $\Phi_{UC,g}$:

$$\Phi_{UC,g} = \Phi_r \frac{F_{UC}}{F_r} \frac{(1-10^{-A_r})}{(1-10^{-A_{UC}})} \frac{\eta_{UC}^2}{\eta_r^2} \quad \text{(S5)}$$

Here, *UC* and *r* denote upconversion and reference sample, respectively. *F* is the integrated emission intensity, *A* the absorption at 405 nm, and η the refractive index of the solvent. Except for the fitting procedure no additional corrections for reabsorption were invoked, even though the UC sample absorption at the fitting wavelength was non-negligible. The presented values of $\Phi_{UC,g}$ are, thus, potentially somewhat underestimated. The procedure for calculating Φ_{UC} was

identical, but the high-energy end of the spectrum (of which parts were typically reabsorbed) was not accounted for (Figure S5, red spectra). Note that the employed definition for $\Phi_{UC,g}$ gives a theoretical maximum of 50%.

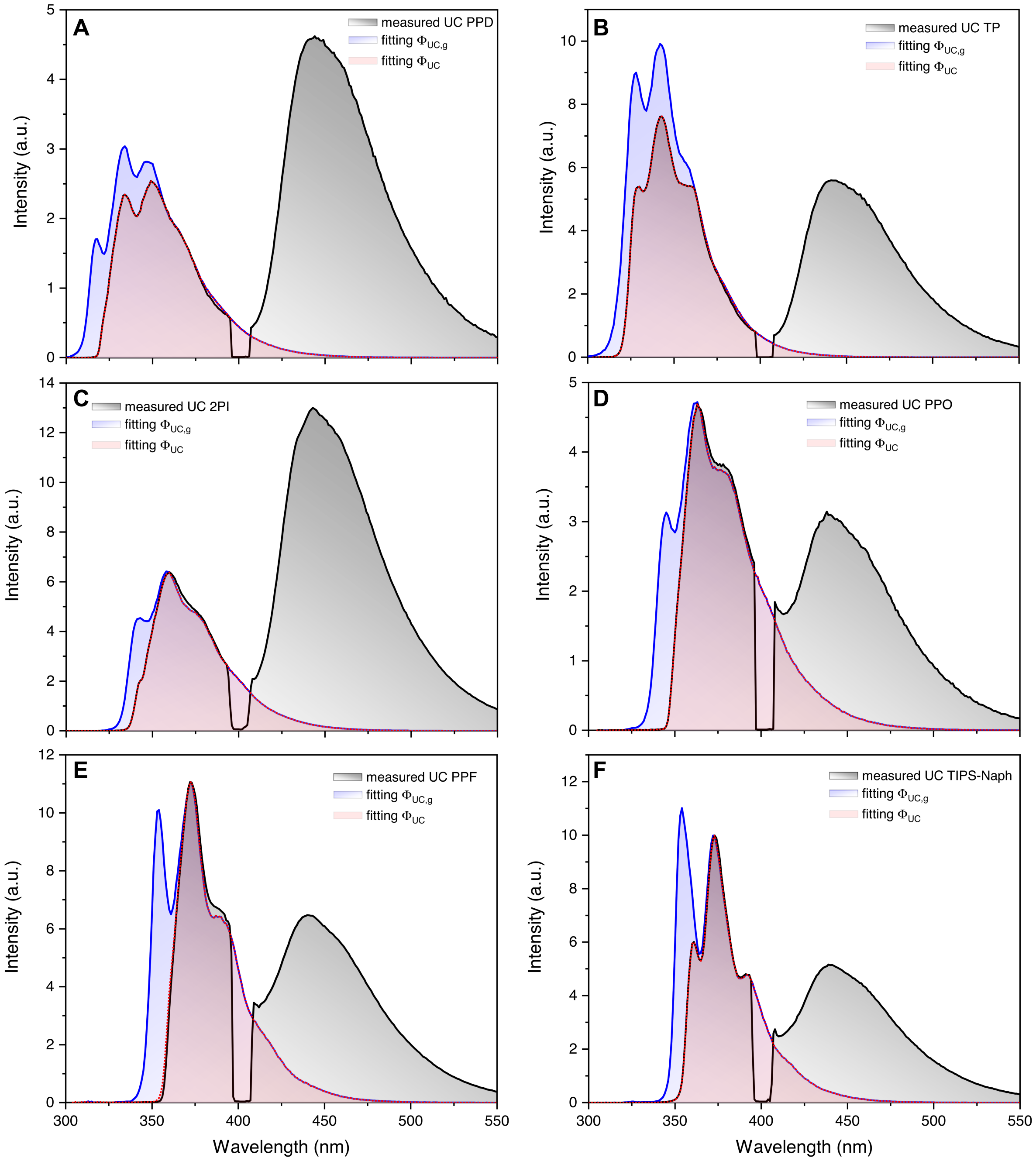

Figure S5. Fitted UC spectra to evaluate $\Phi_{UC,g}$ (blue) and Φ_{UC} (red) from the measured spectra (black). (A) PPD, (B) TP, (C) 2PI, (D) PPO, (E) PPF, and (F) TIPS-Naph.

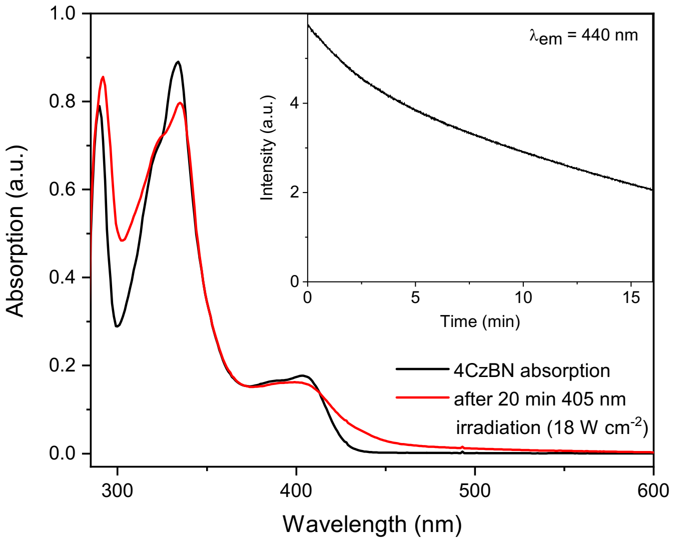

Figure S6. Degradation of 4CzBN.

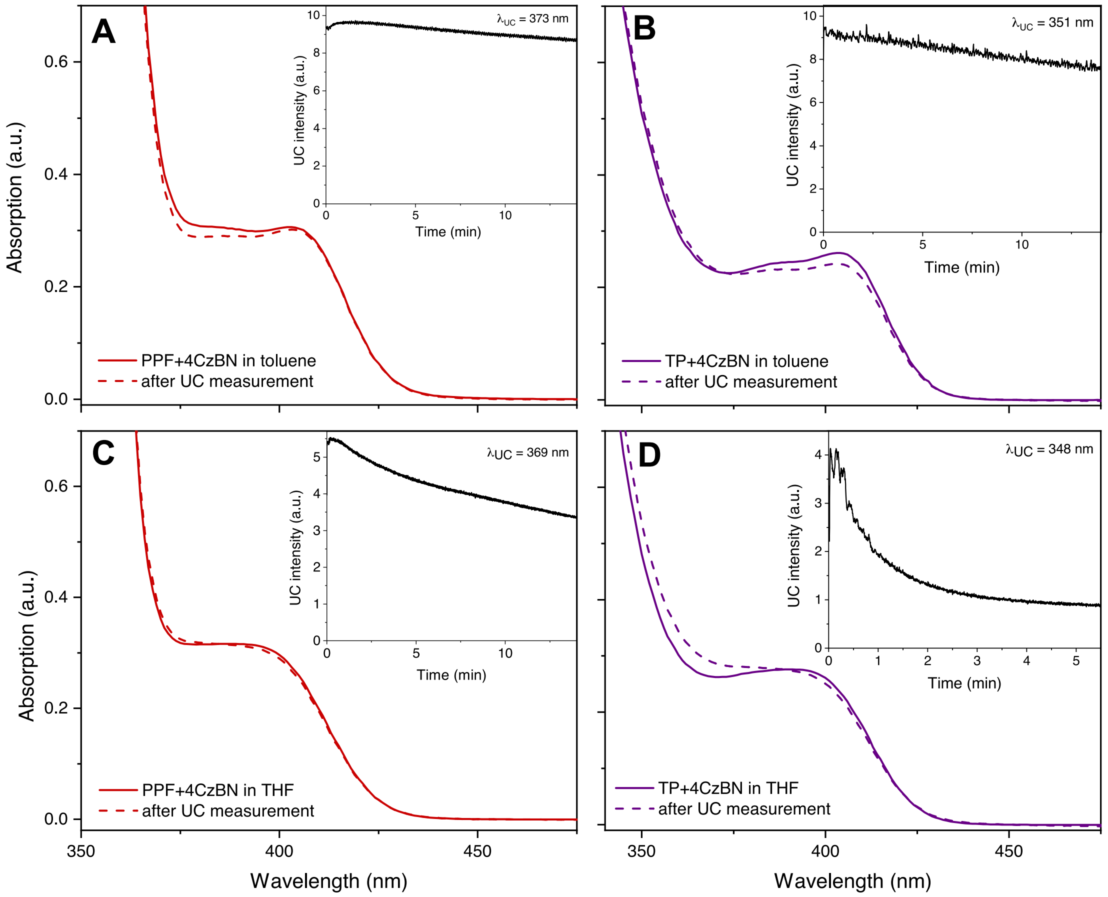

Figure S7. Absorption before (solid) and after 30 minutes (dashed) of 405 nm irradiation at 18 W cm^{-2}. Inset shows the temporal evolution of the UC emission. (A) 4CzBN+PPF in toluene. (B) 4CzBN+TP in toluene. (C) 4CzBN+PPF in THF. (D) 4CzBN+TP in THF.

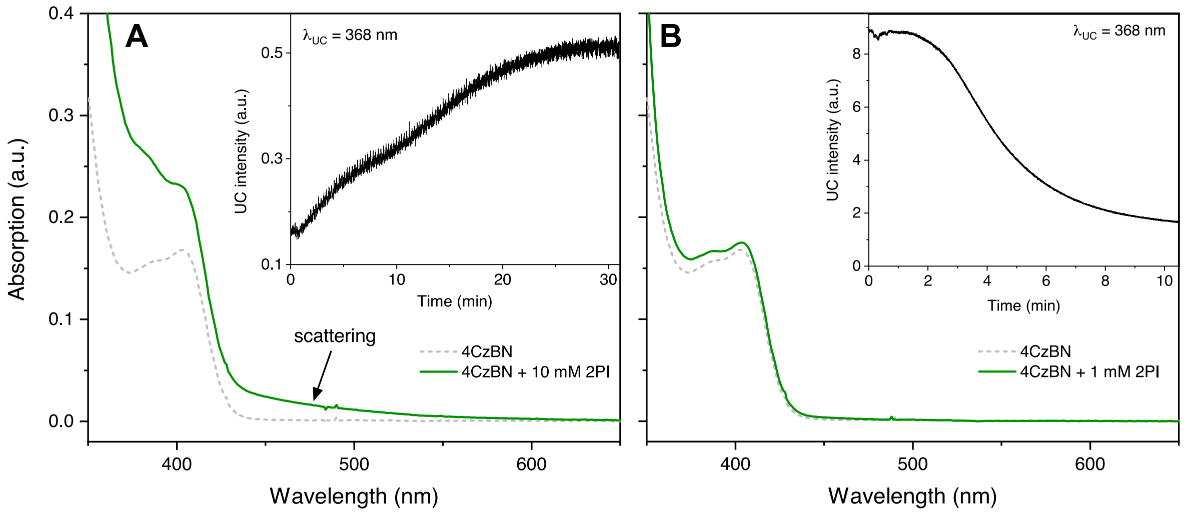

Figure S8. Absorption of 4CzBN with (solid green) or without (dashed grey) (A) 10 mM 2PI, or (B) 1 mM 2PI. Inset: rise/decay of UC emission signal for 2PI probed at 368 nm.

Table S1. Internal UC quantum yields of 25 μM 4CzBN + 10 mM (1 mM for TIPS-Naph and 2PI) annihilator upon 405 nm excitation, measured in deoxygenated THF.

	PPD	TP	2PI	PPO	PPF	TIPS-Naph
$\Phi_{UC,g}$	0.016	0.073	0.021	0.119	0.113	0.141

2.4 Determination of k_T, k_{TTA}, and I_{th} using time-resolved emission

In this section all samples consist of 10 mM annihilator + 25 μM 4CzBN if not stated otherwise (1 mM for 2PI and TIPS-Naph). Time-resolved emission measurements were performed for each annihilator at different excitation intensities, and the measurements were fitted to normalized data using Equation S6 (Equation 2 in the main text). A global fitting procedure was used where β was allowed to vary between measurements, but τ_T was shared globally.

$$I(t) \propto [{}^3A^*]^2 = ([{}^3A^*]_0 \frac{1-\beta}{exp(t/\tau_T)-\beta})^2 \quad \text{(S6)}$$

Higher excitation intensity leads to a higher $[{}^3A^*]_0$ which in turn affords more efficient TTA, leading to a higher value of β (as defined in Equation 3 of the main text). The resulting fittings and obtained triplet lifetimes are presented in Figure S9.

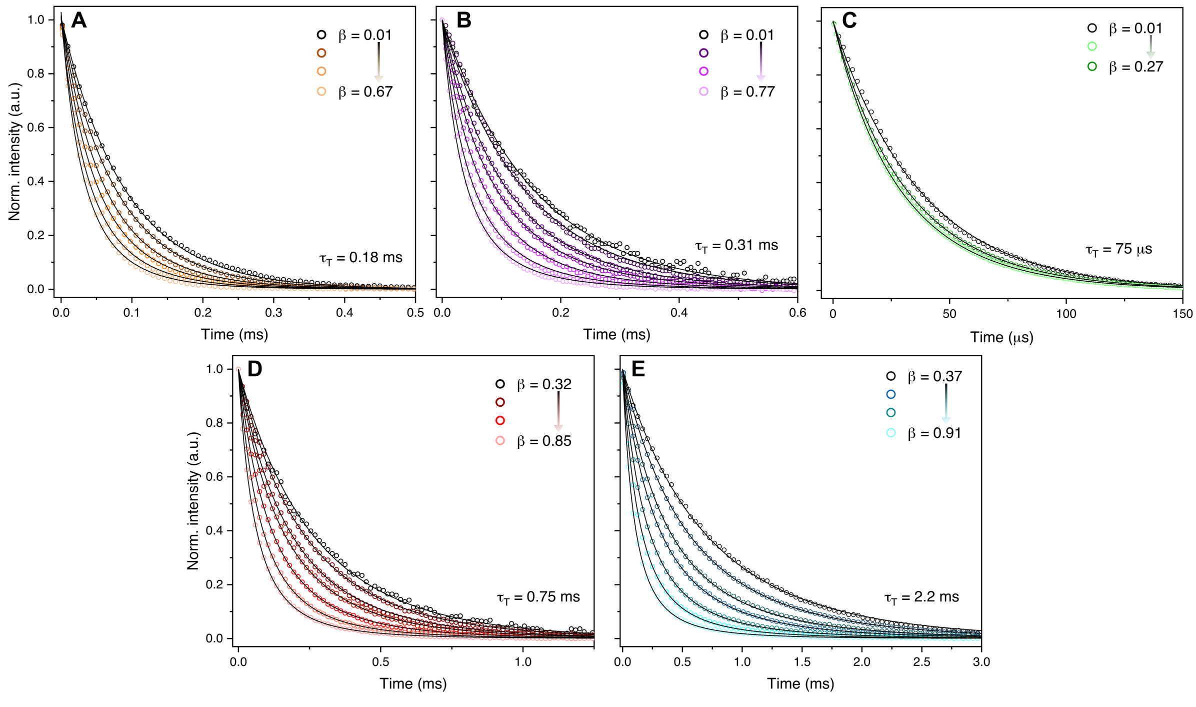

Figure S9. Triplet lifetime determination of annihilator compounds. Some measurements are omitted for clarity purposes. (A) PPD, (B) TP, (C) 2PI, (D) PPF, and (E) TIPS-Naph.

As stated in the main text, by using a 405 nm modulated continuous wave laser diode coupled to a pulse generator we could control the excitation time such that the UC emission had reached a quasi steady-state value before the pulse was turned off and the emission started decaying (Figure 3A in the main text). Assuming that the initial annihilator triplet concentration

equals that of the steady-state triplet concentration ($[^3A^*]_0 = [^3A_{SS}]$) one gets the following equation system with two unknowns: k_{TTA} and $[^3A_{SS}]$.

$$\begin{cases} k_{exc}\Phi_{TET} = 2k_{TTA}[^3A_{SS}]^2 + k_T[^3A_{SS}] \\ \beta = 2k_{TTA}[^3A_{SS}]/(2k_{TTA}[^3A_{SS}] + k_T) \end{cases} \tag{S7}$$

The excitation rate, k_{exc} (M/s), was calculated using Equation S8.

$$k_{exc} = \frac{I_{EX}\lambda(1-10^{-A})}{hcN_AV_{exc}} \tag{S8}$$

Here, I_{EX} is the excitation intensity (W), λ is the excitation wavelength (m), A is the sample absorption at λ, h is Planck's constant (Js), c is the speed of light (m/s), N_A is Avogadro's number (1/mol), and V_{exc} is the excitation volume (dm^3).

To determine k_{TTA} the measurements from Figure S9 (and Figure 3B of the main text) were used, but with β being evaluated in terms of k_{TTA} and k_T, which were shared globally during the fitting procedure. To avoid numeric instability, i.e. to avoid that either Equation S6 or the β expression in Equation S7 approached zero, only the measurements that yielded $0.3 < \beta < 0.7$ during the initial determination of τ_T were used when determining k_{TTA}. Performing the fitting with only the measurements fulfilling this criterion yielded virtually identical τ_T as when using all traces, confirming the validity of this approach. Only for 2PI did we use measurements yielding $\beta < 0.3$ when determining k_{TTA}, simply because $\beta < 0.3$ for all excitation intensities.

The threshold excitation intensity, I_{th}, was determined by evaluating at which excitation intensity β reaches 0.5. The results are shown in Figure S10. The results from the conventional way of determining I_{th} using steady-state measurements are shown in Figure S11 for comparison. Most systems investigated does not fully reach the linear regime in our experimental setup, and fits to slope 1 and 2 become somewhat random. Still, the accordance between I_{th} values determined by the two different methods is acceptable. 2PI displayed no evident transition from the quadratic to the linear regime (Figure S11C), and also at the highest excitation intensity β remained below 0.3. No value for I_{th} could, thus, be determined for 2PI, but the actual value (>25 W cm^{-2}) is orders of magnitude too high to be of interest for solar applications.

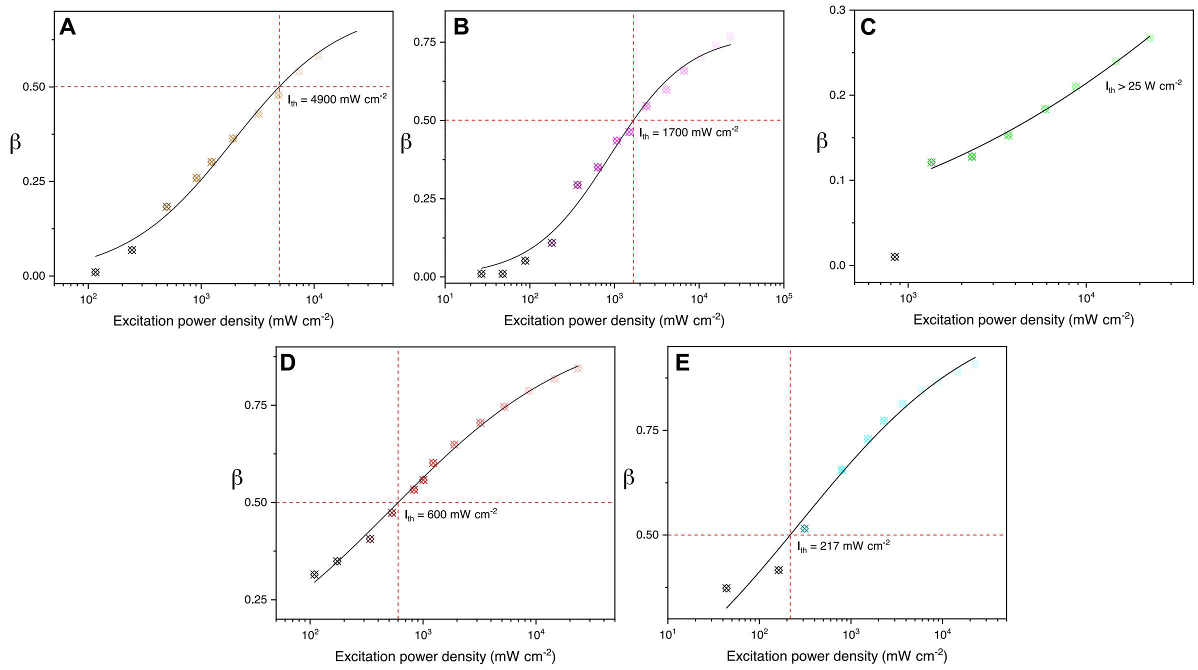

Figure S10. Excitation threshold intensities evaluated at β = 0.5. (A) PPD, (B) TP, (C) 2PI, (D) PPF, and (E) TIPS-Naph. Solid lines are included as guides to the eye.

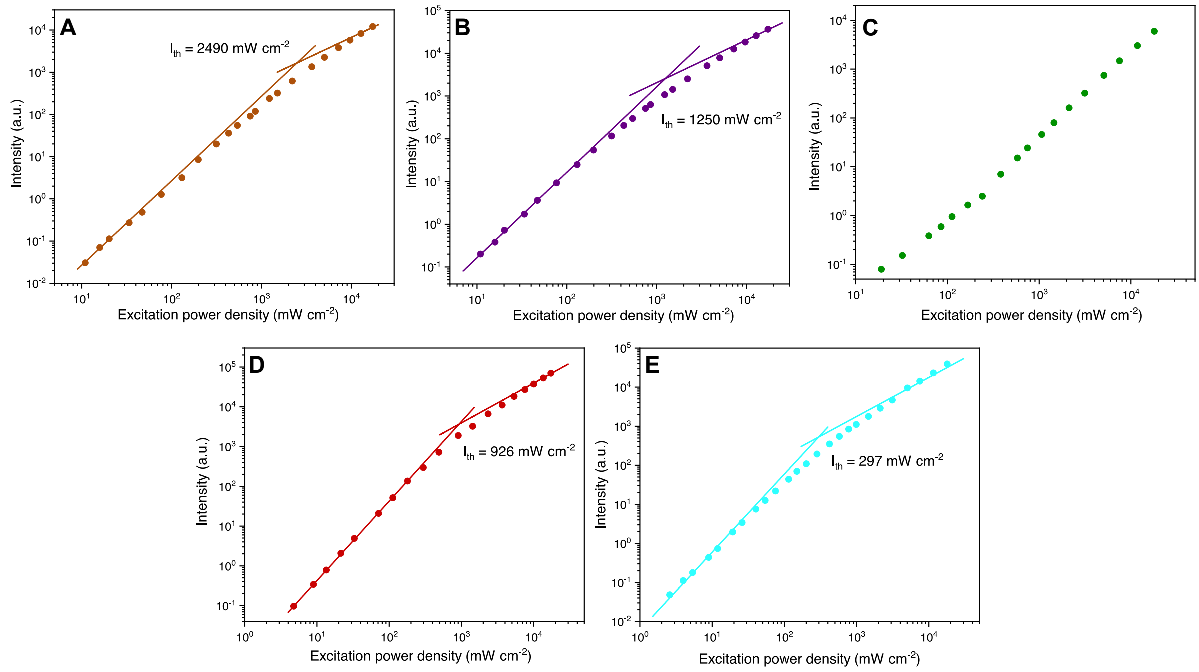

Figure S11. Excitation threshold intensities evaluated using the conventional method with slopes fitted to 1 and 2. (A) PPD, (B) TP, (C) 2PI, (D) PPF, and (E) TIPS-Naph.

2.5 TIPS-Naph contamination

Following the synthesis of TIPS-Naph in accordance with literature procedures,[1] a contamination was discovered. It was not detectable by examination of NMR spectra (Figures S13, S14), only by means of spectroscopy. The contamination was fluorescent and absorbed at 405 nm, the excitation wavelength used during UC experiments. Following several recrystallization cycles the contamination was almost entirely removed but was still evident in the absorption spectrum of 10 mM TIPS-Naph, see Figure S12A.

UC measurements of TIPS-Naph prior to the discovery of the contamination resulted in distorted absorption and UC spectra and relatively low Φ_{UC}, at least partially caused by parasitic absorption of the excitation light by the contamination. Following recrystallization these features were substantially diminished and Φ_{UC} went up. At 10 mM features of fluorescence from the contamination were still present during UC experiments (most evident at approx. 417 nm), causing us to opt for a lower concentration of 1 mM. At this concentration the contamination could not be detected, and the reabsorption of UC emission was also mitigated (Figure S12C).

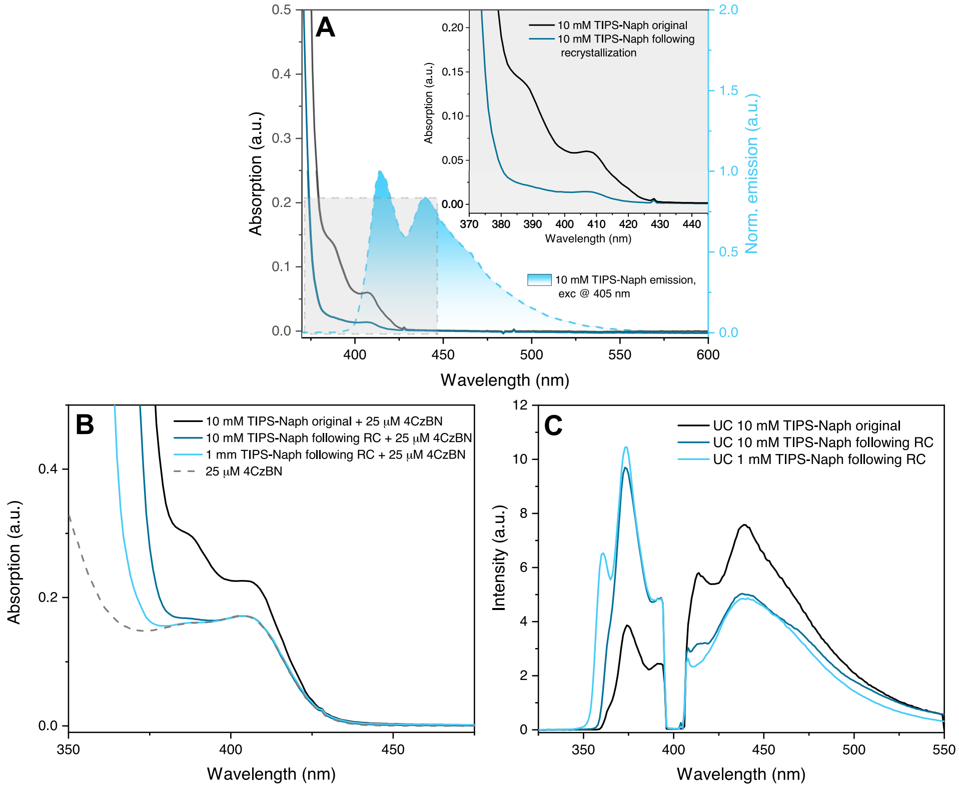

Figure S12. (A) Absorption and fluorescence (λ_{exc} = 405 nm) of TIPS-Naph before and after additional cycles of recrystallization (RC). The fluorescence is originating from the contamination absorbing at around 410 nm. (B) Absorption of UC samples containing TIPS-Naph before and after RC. (C) UC spectra containing TIPS-Naph before and after RC. The spectral shape of prompt fluorescence from 4CzBN is perturbed at 10 mM even after RC.

3. Density Functional Theory Calculations

Table S2. (a) Calculated vertical excitation energies for S_0 optimized geometry (RB3LYP/6-31+G(2d,p)), or (b) experimentally determined energies.

	S_1 (eV)	T_1 (eV)	T_2 (eV)
**PPD**	3.97[a]/3.99[b]	2.85[a]/2.82[b]	3.31[a]
**TP**	4.21/3.98	3.04/2.62	3.62
**2PI**	3.91/3.71	2.33/2.22	3.66
**PPO**	3.64/3.67	2.50/2.40	3.30
**PPF**	3.48/3.59	2.31/2.28	3.28
**TIPS-Naph**	3.35/3.53	2.08/2.12	3.45

4. Synthesis of TIPS-Naph

1,4-dibromonaphthalene (1.44 g, 5.03 mmol), $Pd(PPh_3)_2Cl_2$ (80.6 mg, 0.115 mmol), CuI (60.4 mg, 0.317 mmol), and PPh_3 (82.0 mg, 0.313 mmol) were dissolved in THF (40 mL) under N_2 atmosphere. After adding diisopropylamine (36 mL) to the solution, the mixture was heated to 100 °C. Subsequently, triisopropylsilylacetylene (3.52 g, 19.3 mmol) was added dropwise and the resulting solution was stirred at 100 °C overnight. After cooling to room temperature, THF and diisopropylamine were removed under reduced pressure. The residue was extracted with $CHCl_3$, dried over Na_2SO_4, filtered, and dried under reduced pressure. The crude product was purified by column chromatography (n-hexane) followed by recrystallization from methanol to give TIPS-Naph (69% yield) with ≈99.9% optical purity. Three further consecutive recrystallisation steps was employed to yield TIPS-Naph with an optical purity of ≈99.99% following the discovery of a fluorescent contamination.

^{1}H NMR ($CDCl_3$): δ (ppm) 8.41-8.38 (m, 2H), 7.63 (s, 2H), 7.61-7.58 (m, 2H), 1.20 (s, 36H), 1.19 (s, 6H). ^{13}C NMR ($CDCl_3$): δ (ppm) 133.01, 129.97, 127.00, 126.44, 121.60, 104.58, 97.48, 18.61, 11.24.

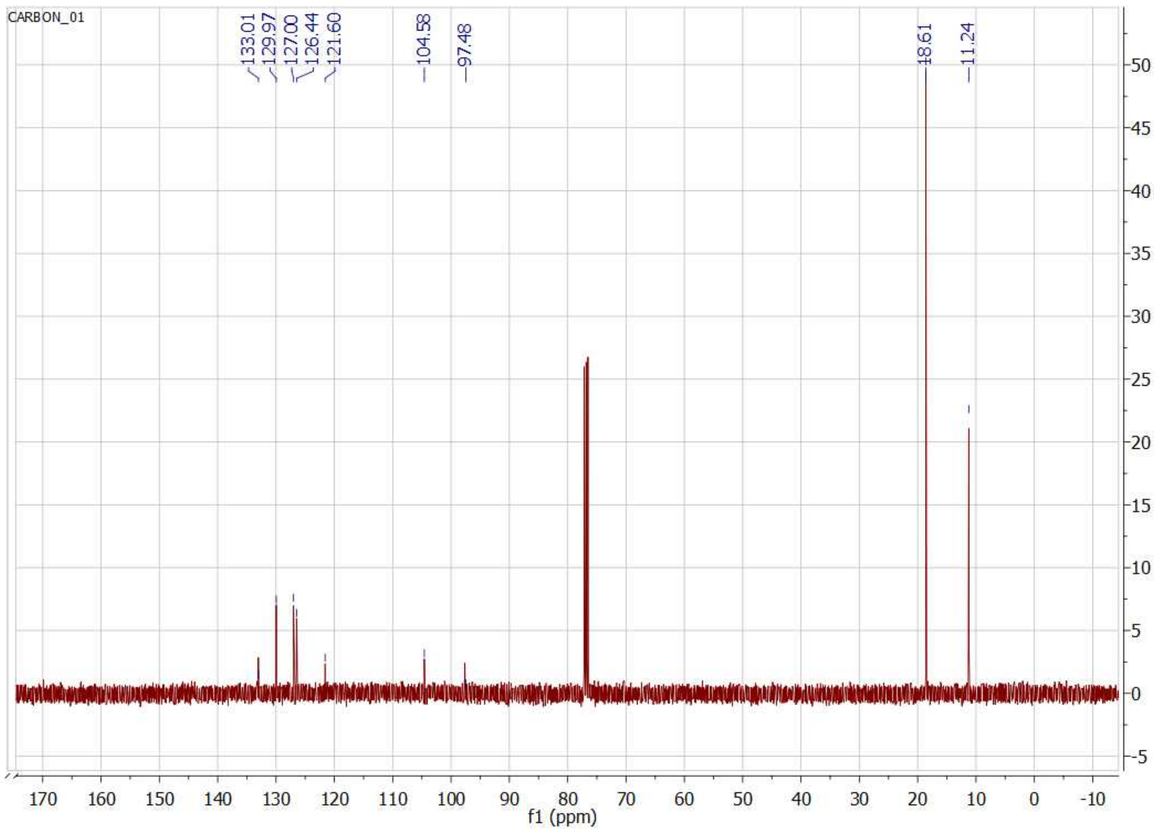

Figure S13. ^{13}C-NMR spectrum of 1,4-bis((triisopropylsilyl)ethynyl)naphthalene (TIPS-Naph).

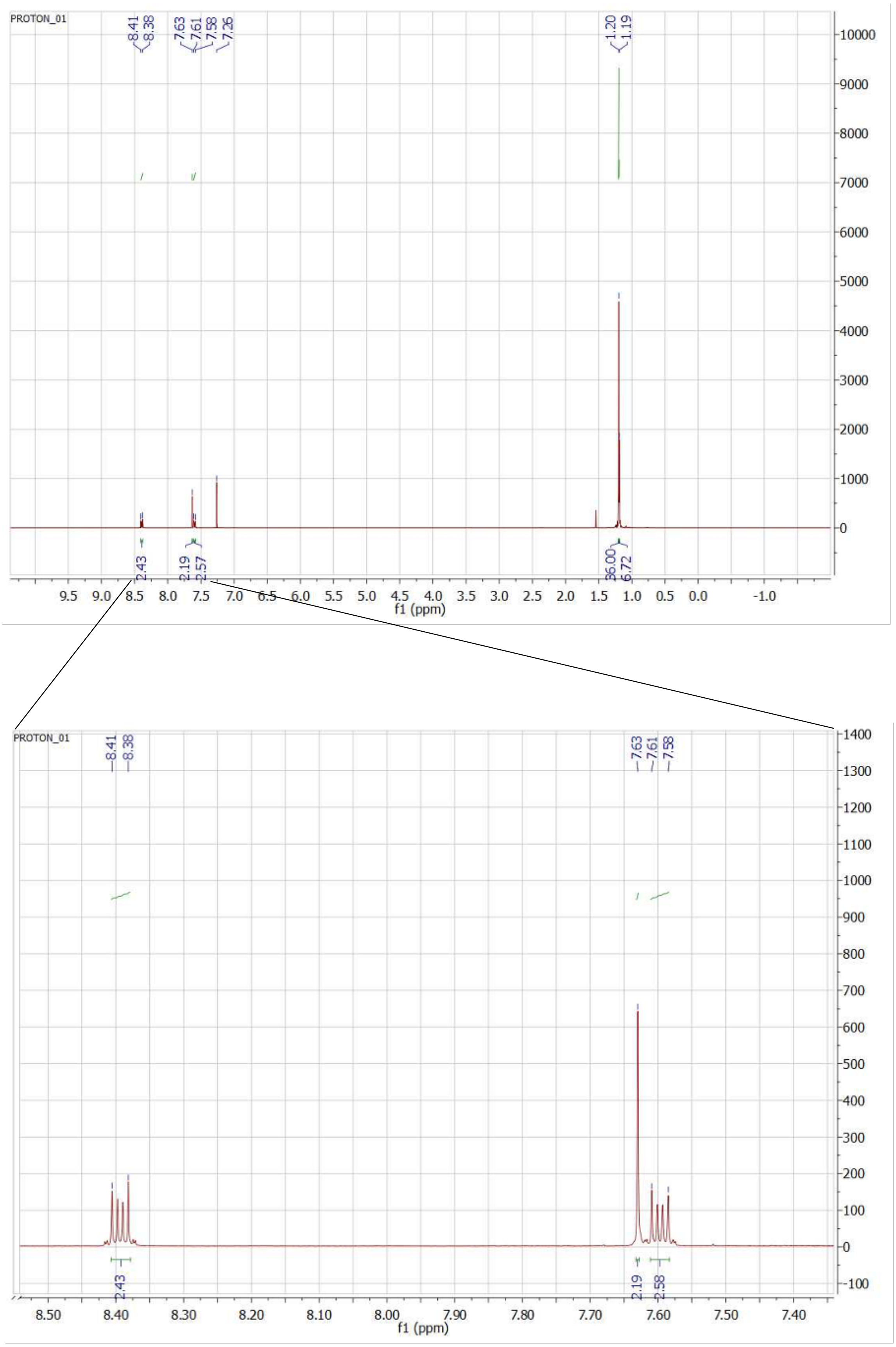

Figure S14. ^{1}H-NMR spectrum of TIPS-Naph.

5. References

1. Harada, N., Sasaki, Y., Hosoyamada, M., Kimizuka, N. & Yanai, N. Discovery of Key TIPS-Naphthalene for Efficient Visible-to-UV Photon Upconversion under Sunlight and Room Light. *Angew. Chemie Int. Ed.* **133**, 144–149 (2021).
2. Berlman, I. Handbook of Fluorescence Spectra of Aromatic Molecules. *Handbook of Fluorescence Spectra of Aromatic Molecules* (Academic Press, 1971).
3. Dias, F. B., Penfold, T. J. & Monkman, A. P. Photophysics of thermally activated delayedfluorescence molecules. *Methods Appl. Fluoresc.* **5**, (2017).
4. Yanai, N. *et al.* Increased vis-to-UV upconversion performance by energy level matching between a TADF donor and high triplet energy acceptors. *J. Mater. Chem. C* **4**, 6447–6451 (2016).
5. Chen, W. *et al.* Red-to-blue photon up-conversion with high efficiency based on a TADF fluorescein derivative. *Chem. Commun.* **55**, 4375–4378 (2019).
6. Zhou, Y., Castellano, F. N., Schmidt, T. W. & Hanson, K. On the Quantum Yield of Photon Upconversion via Triplet–Triplet Annihilation. *ACS Energy Lett.* **5**, 2322–2326 (2020).
7. Olesund, A., Gray, V., Mårtensson, J. & Albinsson, B. Diphenylanthracene Dimers for Triplet–Triplet Annihilation Photon Upconversion: Mechanistic Insights for Intramolecular Pathways and the Importance of Molecular Geometry. *J. Am. Chem. Soc.* **143**, 5745–5754 (2021).
8. Würth, C., Grabolle, M., Pauli, J., Spieles, M. & Resch-Genger, U. Relative and absolute determination of fluorescence quantum yields of transparent samples. *Nat. Protoc.* **8**, 1535–1550 (2013).
